# Materials and Systems for Solar-Driven Interfacial Evaporation: From Material Design to System Integration and Engineering Applications

**DOI:** 10.3390/nano16120767

**Published:** 2026-06-18

**Authors:** Xiao Zhang, Tieling Zhang

**Affiliations:** School of Engineering, Faculty of Engineering and Information Sciences, University of Wollongong, Wollongong, NSW 2522, Australia; xz994@uowmail.edu.au

**Keywords:** solar-driven interfacial evaporation, water purification, photothermal conversion, synergistic management, energy–water coproduction

## Abstract

Solar-driven interfacial evaporation (SIE) has emerged as a transformative, off-grid technology that confines heat at the air–liquid interface, enabling high-efficiency vapor generation for decentralized water purification. Here, we present a comprehensive and critical review of the field, tracing its evolution from fundamental photothermal principles to integrated multifunctional systems. We first elucidate the thermodynamics of interfacial heat localization and the resultant enhancement in evaporation efficiency. We then systematically analyze material innovation strategies—including broadband-absorbing photothermal agents and tailored evaporator architectures—designed to overcome persistent challenges such as salt crystallization, fouling, and thermal losses. Moving beyond freshwater production, we highlight emerging pathways for extending SIE platforms toward water–energy cogeneration, selective resource recovery, and zero-liquid-discharge wastewater treatment. We further identify and objectively assess the key bottlenecks that currently hinder the transition from laboratory-scale prototypes to real-world deployment, with a focus on long-term material robustness under harsh environments, adaptability to fluctuating water chemistries, and techno-economic viability. Finally, we outline forward-looking research directions, including stimulus-responsive smart evaporators, elucidation of multi-field coupling mechanisms, and the establishment of standardized performance evaluation protocols. This review aims to provide both a tutorial for newcomers and a critical assessment for experienced researchers, offering a balanced perspective on the current state-of-the-art and a roadmap for translating SIE from academic research into sustainable, impactful technologies.

## 1. Introduction

### 1.1. Global Water Stress and the Need for Sustainable Solutions

The sustainable supply of freshwater is facing unprecedented pressure worldwide. Although water covers most of the Earth’s surface, easily accessible freshwater accounts for less than 2.5% of the total water volume and is distributed very unevenly [1,2,3]. This strain arises from a complex interplay of population growth, economic expansion, and climate change, leaving billions of people across the globe experiencing varying levels of water scarcity—a critical factor that intensifies social inequality and regional conflicts [4,5]. The crisis takes two main forms: “physical water scarcity”, where natural water resources are inadequate to meet demand [6], common in arid regions; and “economic water scarcity”, where water is physically available but inaccessible due to insufficient infrastructure and management, a frequent challenge in developing areas [7,8,9]. Climate change exacerbates the situation by altering rainfall patterns and increasing the occurrence of extreme droughts, further disrupting the global hydrological cycle and heightening water security risks [10,11].

To address this crisis, attention has turned to the planet’s largest water reserves—seawater and brackish water—through desalination [12,13]. Currently, commercialization relies mainly on conventional thermal and membrane-based technologies such as multi-stage flash distillation and reverse osmosis [14,15]. Although centralized desalination plants support water supply in many coastal areas, they come with inherent and major limitations: high energy consumption, frequent reliance on carbon-intensive power, dependence on large-scale grid infrastructure and piping networks, substantial capital and operational costs, and environmental issues such as brine disposal [16,17]. As a result, these conventional approaches are often economically and practically unsuitable for delivering decentralized, affordable water solutions in widespread inland brackish water zones, remote communities, or emergency situations, creating a significant gap in global water security [18,19].

Therefore, developing decentralized freshwater production technologies that are low-carbon, energy-efficient, modular, and easy to deploy is crucial for building a resilient water security framework [20]. An ideal solution would directly harness renewable energy, shifting the paradigm from centralized distribution to distributed production [21]. Solar energy, given its abundance, accessibility, and sustainability, stands out as the ideal power source for this next-generation desalination strategy [22]. Technologies that use sunlight directly to generate freshwater offer the potential not only to eliminate reliance on fossil fuels and centralized power grids, thereby achieving truly “zero-carbon” water production, but also to meet a wide range of needs, from household to community scale, through flexible modular designs. These features offer a promising new pathway for addressing the global water crisis [23].

A leading contender in this field is solar-driven interfacial evaporation [24]. Its core innovation moves away from the inefficient approach of heating the entire water volume, as seen in traditional solar distillation [25]. Instead, through “interfacial heat localization”, solar absorption and heat generation are confined to a thin layer of water at the liquid–air interface, greatly reducing thermal losses and theoretically enabling a much higher energy conversion efficiency than conventional methods [26]. This feature, together with the potential for simple structures made from abundant materials, makes SIE an ideal candidate to bridge the technological gap between large-scale plants and decentralized, small-scale users. Advancing this technology from laboratory research to practical application holds considerable strategic importance for tackling global water challenges [27].

### 1.2. Fundamental Principles and Historical Evolution of Solar-Driven Interfacial Evaporation Technology

The journey toward efficient solar desalination has witnessed a remarkable evolution, transitioning from simple solar stills to today’s sophisticated interfacial systems [28]. Traditional solar distillation operates on the principle of bulk heating, where sunlight penetrates and warms the entire water volume. This method is inherently limited by significant thermal losses to the environment and the high heat capacity of water, leading to low energy efficiency, typically under 50%, and a constrained daily freshwater output per unit area [29]. For many years, this fundamental drawback restricted the practical application of solar desalination [30]. A significant step forward came with the development of photovoltaic–thermal (PV–T) hybrid systems, which integrate solar electricity generation with the utilization of waste heat for distillation [31]. While improving overall energy use, these systems introduce complexity, incur high costs from photovoltaic panels, and demonstrate sensitivity to water quality and operating temperature, making them less suitable for robust, standalone water production [32].

A true paradigm shift emerged with the conception and validation of solar-driven interfacial evaporation (SIE) [25]. Its core innovation lies in the principle of “interfacial heat localization”. In contrast to bulk heating, SIE systems utilize a specially engineered floating photothermal structure. This design efficiently absorbs sunlight and converts it into heat precisely at the liquid–air interface, creating a localized hot zone within a thin surface layer of water [33]. This approach strategically minimizes three primary heat loss pathways: (1) conductive loss to the underlying water is reduced by a thermally insulating substrate; (2) convective loss to the surrounding air is mitigated by localizing heat at the surface; and (3) radiative loss is diminished due to the small, heated area. Consequently, the input energy is concentrated primarily on vaporizing water at the interface, leading to a dramatic increase in thermal efficiency [34].

The modern era of SIE research is often traced to the landmark 2016 work by Zhou et al., which demonstrated a double-layered evaporator using carbon foam [35]. This system achieved a remarkable solar-thermal conversion efficiency of approximately 90% under one-sun illumination, conclusively proving the power of interfacial heating. This breakthrough ignited widespread global research activity. Subsequent progress has advanced along two interconnected fronts: material innovation and structural/mechanistic design [36]. Early efforts focused on exploring a broad spectrum of high-performance photothermal materials, including plasmonic metals (e.g., Au, Al nanoparticles), carbon-based materials (e.g., graphene, CNTs, biochar), semiconductors, and polymers [37]. Concurrently, structural engineering evolved from simple 2D floating sheets to more sophisticated 3D and Janus architectures designed to enhance light absorption, water transport, and vapor release [38,39]. A critical realization emerged that long-term performance depends not merely on high evaporation rates, but also on effective salt rejection and fouling resistance. This insight spurred innovative strategies for managing the interplay among water, salt, and heat, shifting the field’s focus from peak laboratory performance to solving practical durability challenges [40].

Today, SIE stands as a distinct and promising technological pathway. It fundamentally differs from both passive solar stills and active PV–T systems through its elegant, efficiency-centric design principle [41]. The historical progression reveals a clear transition from using solar energy as a generalized heat source to precisely engineering its conversion and confinement at the critical phase-change interface. This evolution has transformed SIE from a novel concept into a rapidly advancing platform for sustainable freshwater generation, with contemporary research increasingly aimed at system integration, scalability, and real-world operation [42].

### 1.3. Key Performance Metrics for Technology Development

Translating SIE technology from laboratory prototypes to practical applications requires a rigorous and standardized framework for performance evaluation. A comprehensive set of key performance indicators (KPIs) is essential not only for comparing different designs but also for steering optimization efforts toward real-world viability [43]. These metrics collectively define the efficiency, durability, scalability, and economic feasibility of SIE systems, shifting the focus beyond the initial, narrow emphasis on evaporation rates under ideal conditions [44].

The most fundamental KPIs quantify the core photothermal conversion and vapor generation processes [45]. The evaporation rate, typically expressed in kg·m^−2^·h^−1^ under one-sun illumination, directly reflects freshwater production capacity [46]; however, short-term measurements alone can be misleading. The key thermodynamic metric is the solar-to-vapor conversion efficiency (η), calculated as η = (ṁ·h_lv_)/P_in_, where ṁ is the evaporation rate, h_lv_ is the effective enthalpy of vaporization, and P_in_ is the incident solar power density [47]. High efficiency values often exceeding 90% under controlled laboratory conditions demonstrate successful thermal localization. These outcomes rely on the material’s high broadband solar absorptance and low mid-infrared thermal emittance, which maximize light harvesting and minimize radiative heat loss, respectively [48].

Beyond these core energy-conversion metrics, long-term performance under realistic operating stresses distinguishes promising prototypes from deployable technologies. Stability must be assessed over extended continuous operation (e.g., >100–200 h) and through repeated day–night cycles to evaluate degradation [49]. For desalination applications, salt rejection capability is critical and can be assessed by the duration of stable evaporation, the control of salt crystallization, and the system’s autonomous recovery capability. Similarly, resistance to biofouling and chemical degradation in varied water compositions largely determines operational lifespan [50]. For integrated systems, overall water-production efficiency, which accounts for condensation and collection losses, provides a more realistic measure of actual yield than evaporation rate alone [37]. In multifunctional configurations, an energy utilization factor that evaluates all useful outputs (vapor, electricity, etc.) against total solar input offers a holistic performance perspective [51].

Ultimately, the practical deployment of SIE depends on economic and environmental viability [52]. Material and manufacturing cost per unit area indicates scalability, while a projected levelized cost of water, incorporating capital expense, lifetime, and operational inputs, provides a preliminary economic outlook [53]. An early-stage environmental assessment should examine sustainability from material sourcing through to end-of-life disposal. Importantly, these metrics are interrelated and often involve trade-offs; for example, maximizing the evaporation rate through intricate 3D structures may increase costs and complicate salt management. Thus, optimizing SIE systems is inherently a multi-objective challenge [54]. The field is increasingly progressing toward reporting a consolidated set of KPIs under standardized test conditions, including specified light intensity, water salinity, and ambient parameters to enable fair comparisons and transparently evaluate the technology’s readiness to transition from laboratory research to real-world application [55].

### 1.4. Framework and Objectives of This Work

The field of solar-driven interfacial evaporation has progressed from its early focus on photothermal materials, yet its path toward practical application is hindered by a research landscape dominated by incremental advances under idealized laboratory conditions [56]. This has widened the gap between lab-scale prototypes and the demands of scalable, durable, and economically viable technology. To close this gap, this review advocates a paradigm shift—from optimizing individual components to pursuing system-level synergistic design [54]. We organize our analysis around the coupled physics of energy and mass transfer across scales, from nanoscale interfacial water regulation to macroscale water harvesting. Furthermore, we critically assess material scalability and integrate discrete water networks with fixed infrastructure. The overarching aim is to provide a coherent, critical, and forward-looking blueprint that bridges laboratory innovation and practical decentralized desalination [57].

**Figure 1:**
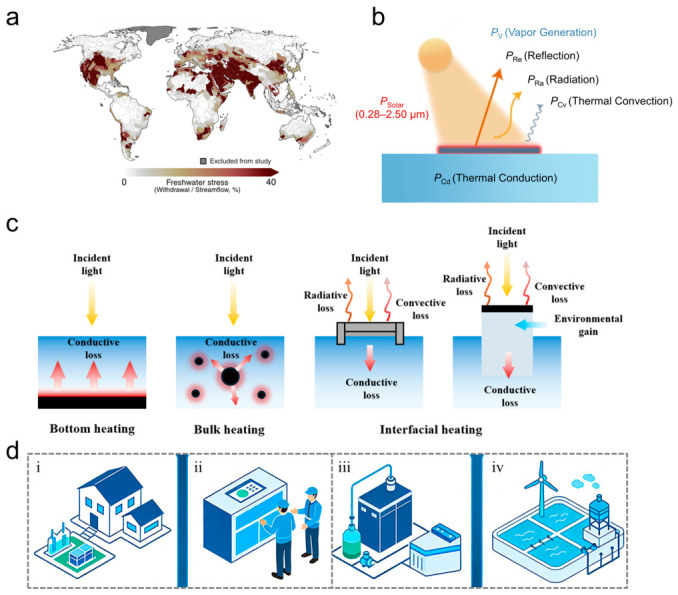
(**a**) Global freshwater stress, derived from freshwater withdrawal and streamflow datasets. Reprinted from Ref. [61]. (**b**) Multiple heat losses occurring in solar-powered evaporators with solar radiation as the sole energy input. Reprinted with permission from Ref. [37]. Copyright 2025 American Chemical Society. (**c**) Energy nexus during different solar steam generation processes [43]. (**d**) All-scenario applications of solar interfacial evaporation (SIE): i. decentralized water supply for remote communities or islands; ii. portable water production devices for emergency relief; iii. high-salinity wastewater treatment; iv. aquaculture-electricity co-generation, along with other multi-functional scenarios.

To ground this framework in the broader context of global water challenges and the physical principles of solar evaporation, Figure 1 provides an integrated overview. Figure 1a illustrates the widespread freshwater stress driven by the imbalance between withdrawal and natural streamflow, underscoring the urgency for decentralized water production. Figure 1b,c breaks down the multiple heat losses inherent in solar-powered evaporators and the energy nexus among different steam generation processes, highlighting the thermodynamic constraints that any practical system must navigate. Finally, Figure 1d maps the all-scenario applications of solar interfacial evaporation (SIE), from decentralized supply and emergency relief to high-salinity wastewater treatment and aquaculture-electricity cogeneration, setting the stage for the technology’s multifunctional potential discussed throughout this review.

The conceptual framework of this review rests on a central premise: the ultimate performance and viability of an SIE system are dictated by the integrated management of water, heat, and salt transport [58]. Excellence in one domain, such as achieving exceptional photothermal conversion, can be easily undermined by failure in another, such as salt accumulation. Accordingly, our analysis is designed to dissect and reconnect these core physical processes across all scales of design [59]. Moving beyond optical metrics, we critically assess how material engineering, through tailored surface chemistry, strategic hybridization, and nanoscale structuring, can be intentionally designed to simultaneously regulate wettability, resist scaling, and even introduce secondary functions like catalysis, thereby laying the groundwork for integrated system performance [37].

Here, we provide a systematic evaluation of structural innovations, from 2D films to 3D and Janus configurations, focusing on their effectiveness in spatially coordinating the frequently competing flows of water, heat, and salt ions [43]. This establishes the crucial link where material potential is realized as engineered function. Building on this foundation of optimized unit design, Section 4 expands the view to system integration and multifunctionality. We examine engineering strategies for modular and array-based scaling, and critically evaluate the coupling of SIE with complementary processes, particularly water-electricity cogeneration, to analyze the energy balances, added complexities, and overall benefits of such integrated systems [60].

Section 5 offers a critical review of the current lack of standardized evaluation protocols and argues for the adoption of stability-focused, application-relevant performance metrics. We further provide a clear-eyed discussion of economic feasibility and environmental impact through techno-economic analysis and life cycle assessment. Section 6 discusses performance evaluation standards, practical application challenges, and outlook. Finally, the review concludes by synthesizing these insights into a forward-looking roadmap, as shown in Section 7, identifying key interdisciplinary research frontiers that must be advanced to transition SIE from a compelling laboratory concept to a pragmatic technology capable of addressing global water challenges.

The overarching aim of this review is to provide a coherent, critical, and forward-looking blueprint for applied SIE research. By integrating knowledge across materials science, thermal engineering, and system design, we seek to equip researchers with a unified framework that prioritizes synergy, durability, and scalability. It is our intent that this work will not only summarize the current state of the field, but more importantly, offer a strategic vision for its future direction, promoting the development of robust, solar-powered water-production systems that can truly function in the complex and variable conditions of the real world.

## 2. Literature Search and Analysis

To ensure the systematicity, comprehensiveness, and objectivity of this review, a standardized and replicable literature search and screening workflow was strictly implemented, targeting the core scope of this work. This section details the retrieval strategy, database selection, keyword strategy, stepwise screening process, and final inclusion criteria of the references, establishing a rigorous literature foundation for the subsequent technical analysis and forward-looking discussion.

### 2.1. Databases and Retrieval Scope

The core literature retrieval was conducted based on the Web of Science Core Collection (WoSCC), the most authoritative database for global academic research in the field of materials science and environmental engineering, which covers high-quality peer-reviewed journals including the Nature series, Advanced Materials series, ACS Nano, Journal of Materials Chemistry A, Desalination and other top journals in the relevant field. To ensure comprehensive retrieval, Scopus, Google Scholar, and EI Compendex were used as supplementary databases to avoid missing high-impact pioneering works, critical reviews, and the latest breakthrough research.

The retrieval time range was set from January 2000 to February 2026. This time span covers the early theoretical exploration of traditional solar distillation, the paradigm shift of interfacial heat localization, the landmark breakthrough of modern solar-driven interfacial evaporation (SIE) technology in 2016, and the latest research progress in material innovation, structural optimization, system integration, and engineering application up to the present. Meanwhile, the retrieval was limited to document types of articles and reviews, excluding conference abstracts, patents, book chapters, editorial materials, and retracted papers, to ensure the academic rigor and data reliability of the included literature.

### 2.2. Keyword System and Retrieval Strategy

A multi-level keyword system combined with Boolean logical operators was established for precise retrieval, which strictly aligns with the core framework of this review. The core retrieval formula is as follows:

(“solar-driven interfacial evaporation” OR “solar interfacial evaporation” OR “solar steam generation” OR “interfacial heat localization”) AND (“photothermal material” OR “evaporator structural design” OR “salt rejection” OR “thermal management” OR “system integration” OR “solar desalination” OR “wastewater treatment” OR “engineering application”)

The keyword system is divided into two dimensions: the first dimension is the core subject keywords, which define the research object of SIE technology and its core principle; the second dimension is the thematic extension keywords, which cover the full technical chain of this review from material design, structural optimization to system integration and practical application. This retrieval strategy ensures that the literature obtained covers all the core sections of this review, avoiding the omission of key research progress in any technical link.

### 2.3. Stepwise Literature Screening Process

A three-stage stepwise screening workflow was implemented to filter the initially retrieved literature, with clear inclusion and exclusion criteria for each stage, and the whole process was managed by EndNote 2025 literature management software to ensure traceability and repeatability.

Duplication removal and preliminary screening: A total of 3286 relevant studies were initially retrieved from all databases. After removing duplicate literature through the software and manual cross-checking, 2417 unique studies were retained. Then, the title and abstract were screened for the first time: studies that were completely unrelated to the research scope, such as non-solar-driven evaporation technology, pure photocatalytic research without interfacial evaporation process, traditional bulk solar distillation without interfacial heat localization design, were excluded, and 582 studies were retained after this stage.

Full-text screening and quality evaluation: The full text of the 582 retained studies was read and evaluated one by one. The evaluation criteria included the academic innovation of the research, the integrity and reliability of experimental data, the authority of the journal, and the fit with the core theme of this review. Studies with repetitive research content, low academic quality, incomplete data support, and poor alignment with the technical framework of this review were excluded. Meanwhile, the references cited in high-impact review articles were manually traced to supplement missing pioneering landmark works and key comparative studies.

Final inclusion and classification: After the above screening, a total of 243 core references were finally included as the main reference basis for this review. The selected literature was classified and organized according to the framework of this review, encompassing fundamental principles and mechanisms, photothermal material engineering, evaporator structural design, salt rejection and anti-fouling strategies, thermal management, system integration and engineering application, and technical bottlenecks and prospects. This classification supports a systematic discussion in each section.

## 3. Advancing Photothermal Conversion Materials Toward Multifunctionality

### 3.1. Physical Mechanisms of Photothermal Conversion in Materials

The unparalleled appeal of SIE stems from its elegant simplicity: the direct and efficient transformation of sunlight into localized heat at a water–air interface. This foundational process hinges entirely on the intrinsic ability of photothermal materials to capture photons and convert their energy into lattice vibrations (phonons), resulting in a temperature rise. A deep understanding of the distinct physical mechanisms governing this conversion is paramount for the rational design of next-generation materials, moving beyond empirical discovery toward predictive engineering. These mechanisms are primarily dictated by the electronic band structure and the interaction between incident light and charge carriers within the material [62].

Plasmonic heating, exhibited by noble metal nanoparticles (e.g., Au, Ag) and some heavily doped semiconductors, arises from the collective oscillation of conduction-band electrons when excited by light at a resonant frequency [63]. This phenomenon, known as localized surface plasmon resonance (LSPR), creates intense, confined electromagnetic fields near the nanoparticle surface [64]. The subsequent rapid non-radiative decay of these plasmonic oscillations, primarily via electron–electron and electron–phonon scattering, generates substantial localized heat [65]. The key advantage of this mechanism lies in its spectral tunability; the LSPR peak can be precisely engineered across the visible to near-infrared spectrum by manipulating the nanoparticle’s size, shape, and surrounding dielectric environment. This allows for the design of materials with targeted absorption profiles [66].

For semiconductors with a bandgap energy less than the energy of incident photons, electron–hole pairs are generated upon illumination [67]. These photoexcited carriers thermalize to the band edges through rapid intra-band relaxation, releasing energy as heat [68]. Finally, the carriers recombine through non-radiative recombination pathways, where the energy is transferred directly to the crystal lattice as phonons. Similarly, in carbon allotropes like graphene, carbon nanotubes, and biochar, the broadband absorption is attributed to π-plasmon resonance and the excitation of delocalized π-electrons across a continuum of states. The relaxation of these highly excited electronic systems occurs overwhelmingly through non-radiative channels, making carbon materials exceptionally efficient and broadband photothermal converters [69].

To quantitatively evaluate the overall energy conversion process, the absorbed solar power at the evaporating interface must be balanced against the heat consumed for vaporization and the various loss pathways. The spectrally weighted absorptivity determines the harvested power:

(1) $$\alpha =\frac{\int_{300}^{2500}I(\lambda )A(\lambda )d\lambda }{\int_{300}^{2500}I(\lambda )d\lambda },{\dot{Q}}_{abs}=\alpha {\dot{Q}}_{solar}$$

where ${\dot{Q}}_{solar}=1000\;W\cdot m^{-2}$ under 1 sun illumination, I(λ) is the incident solar spectral irradiance, and A(λ) is the wavelength-dependent absorptance. The steady-state energy balance is:

(2) $${\dot{Q}}_{abs}={\dot{Q}}_{vap}+{\dot{Q}}_{rad}+{\dot{Q}}_{cond}+{\dot{Q}}_{conv}$$

with ${\dot{Q}}_{vap}=m^{\prime \prime }h_{fg}$ ($m^{\prime \prime }$: evaporation mass flux in kg·m^−2^·s^−1^; $h_{fg}$: latent heat of vaporization in J·kg^−1^). The three loss terms are expressed as:

Radiation loss:

(3) $${\dot{Q}}_{rad}=\frac{\sigma (T_{eva}^{4}\;-\;T_{cd}^{4})}{\frac{1}{\varepsilon_{eva}}\;+\;\frac{1}{\varepsilon_{cd}}\;+\;\frac{1}{X}-2}$$

where σ is the Stefan–Boltzmann constant (5.67 × 10^−8^ W·m^−2^·K^−4^), $T_{eva}$ and $T_{cd}$ are the temperatures (K) of the evaporator and condenser, $\varepsilon_{eva}$ and $\varepsilon_{cd}$ their emissivities, and X is the angular coefficient depending on the gap geometry.

Conduction loss:

(4) $${\dot{Q}}_{cond}=\frac{\lambda_{a}}{d_{ag}}(T_{eva}-T_{cd})+\frac{\lambda_{eva}}{\delta_{eva-cs}}(T_{eva}-T_{cs})$$

where $\lambda_{a}$ and $\lambda_{eva}$ are the thermal conductivities (W·m^−1^·K^−1^) of air and the evaporator material, $d_{ag}$ is the air gap distance (m), $\delta_{eva-cs}$ is the evaporator thickness (m), and $T_{cs}$ is the bulk water temperature (K).

Convection loss:

(5) $${\dot{Q}}_{conv}=h_{conv,a}(T_{eva}-T_{cd})$$

where $h_{conv,a}$ is the convective heat transfer coefficient (W·m^−2^·K^−1^) of air.

The solar-to-vapor efficiency is then defined as:

(6) $$\eta =\frac{m^{\prime \prime }h_{fg}}{\alpha {\dot{Q}}_{solar}}$$

These equations provide a quantitative link between material properties (absorptivity *α*, emissivity ε, thermal conductivity λ) and system performance, revealing the physical limits of interfacial solar evaporation and guiding rational material design.

Beyond electronic processes, molecular vibrational heating plays a dominant role in organic polymers and certain semiconductor materials [70]. In polymers like polypyrrole (PPy) or polydopamine (PDA), incident photons are absorbed by molecules, promoting electrons from the highest occupied molecular orbital (HOMO) to the lowest unoccupied molecular orbital (LUMO). The subsequent relaxation of these excited molecules does not primarily emit light (fluorescence), but instead undergoes efficient internal conversion and vibrational relaxation. In this process, the electronic excitation energy is rapidly transferred into various vibrational modes of the molecular skeleton and surrounding matrix, manifesting as heat. Materials leveraging this mechanism often exhibit strong, wide absorption due to complex molecular structures and are prized for their biocompatibility and ease of fabrication [71]. The following table (Table 1) summarizes the key characteristics of these primary photothermal mechanisms:

In practical SIE systems, these mechanisms are seldom exclusive. Advanced photothermal materials often employ hybrid designs to synergize their benefits. For instance, depositing plasmonic nanoparticles on a semiconductor or carbon substrate can combine strong, resonant absorption with broadband thermal generation and enhanced stability. The choice and engineering of the dominant conversion mechanism directly influence critical performance parameters, including the solar absorption spectrum, the ultimate steady-state temperature, and the thermal response time of the evaporator, setting the stage for the multifunctional material engineering strategies discussed in the following sections [72].

Despite their high photothermal conversion efficiency, the practical application of plasmonic nanomaterials (Au, Ag), as well as other emerging materials such as MXenes and metal sulfides (e.g., CuS, MoS_2_), raises critical concerns regarding long-term environmental stability, toxicity, and potential leaching [73,74]. Silver nanoparticles, for instance, are prone to oxidation and the release of biocidal Ag^+^ ions, which can harm aquatic organisms if leached into natural water bodies; gold nanoparticles are more stable but still pose potential ecotoxicity depending on size, shape, and surface functionalization. MXenes gradually oxidize under light irradiation in aqueous environments, forming TiO_2_ and carbon species that may degrade photothermal performance and release unknown degradation products [75]. Metal sulfides can undergo photo-corrosion or chemical dissolution in saline or acidic conditions, leaching toxic metal ions and sulfide species. To date, the SIE literature lacks systematic assessments of accelerated aging, leachate characterization, eco-toxicological testing, and effective mitigation strategies such as protective coatings or embedding in stable polymer matrices. Future research must prioritize these environmental safety aspects to ensure that emerging photothermal materials enable not only high-performance, but also sustainable and ecologically benign water purification [76].

### 3.2. Classical and Emerging Photothermal Materials

The evolution of solar-driven interfacial evaporation (SIE) has been fundamentally propelled by the exploration and engineering of photothermal materials. These materials form the essential interface where sunlight is captured and transformed into heat. Over the past decade, research has progressed from utilizing a handful of classical absorbers to strategically designing a diverse portfolio of materials, each with distinct physicochemical properties tailored for specific functions beyond mere light absorption. This section systematically examines these material families, delineating their intrinsic photothermal mechanisms, evolution, and their role in advancing SIE technology toward practical applications (Table 2).

Plasmonic metallic materials, primarily gold (Au), silver (Ag), and aluminum (Al) nanostructures, represent a classical yet highly tunable category [77]. Their photothermal activity originates from the excitation and subsequent non-radiative decay of localized surface plasmon resonances (LSPRs). The primary advantage lies in the exquisite spectral tunability; the resonance peak can be precisely engineered from the visible to the near-infrared region by controlling the nanoparticle size, shape (e.g., nanospheres, rods, shells), and local dielectric environment [78]. This enables the design of materials with high absorption at specific solar wavelengths. However, the high cost of noble metals and their susceptibility to oxidation, coalescence, or ion leaching in harsh aqueous environments have limited their use in large-scale, durable SIE systems. Recent efforts focus on using earth-abundant alternatives like aluminum, stabilizing noble metal nanoparticles within robust matrices, and designing intricate nanostructures (e.g., core-shell, porous assemblies) to enhance broadband absorption and environmental stability [78].

Carbon-based materials, including graphene, carbon nanotubes (CNTs), carbon black, and biochar-derived carbons, are perhaps the most widely studied classical photothermal agents due to their exceptional attributes [79]. They offer near-perfect broadband solar absorption arising from π–π* transitions and plasmonic effects in a disordered graphitic network. Coupled with excellent thermal conductivity, chemical inertness, low cost, and scalability, carbon materials provide a robust foundation for evaporators [80]. The focus on carbon materials has shifted from demonstrating basic performance to functionalization and structuring. For instance, graphene oxide (GO) sheets can be assembled into hierarchical aerogels with tuned porosity for rapid water transport, while biochar derived from biomass waste offers a sustainable and ultralow-cost pathway. The current challenge lies in further enhancing their water-wicking properties and integrating them into mechanically robust, monolithic structures without compromising their porous networks [81].

Semiconductor materials, such as transition metal oxides/sulfides (e.g., Cu_2_S, Ti_2_O_3_, MoS_2_), MXenes, and covalent organic frameworks (COFs), constitute a rapidly emerging class [82]. Their photothermal conversion typically relies on the non-radiative recombination of photoexcited electron–hole pairs within a narrow or intermediate bandgap. A significant advantage of many semiconductors is their potential for multifunctionality. For example, certain metal sulfides can act as catalysts for degrading organic pollutants simultaneously with evaporation [83]. MXenes combine high photothermal efficiency with metallic conductivity, enabling integrated solar–thermal–electric conversion. COFs offer molecularly precise, porous platforms that can be pre-designed to incorporate specific functional groups for targeted contaminant adsorption. The research frontier for semiconductors involves tailoring their bandgap and surface chemistry for optimal solar harvesting, improving their dispersion and processability, and enhancing their long-term stability in water [84].

Polymeric and organic materials, including polypyrrole (PPy), polyaniline (PANI), polydopamine (PDA), and other conjugated polymers, operate primarily through molecular vibrational heating following photon absorption [85]. They are characterized by good biocompatibility, mechanical flexibility, and facile synthesis/coating processes, allowing them to conform to various substrates. PDA, inspired by mussel adhesion, is particularly notable for forming uniform, hydrophilic coatings on virtually any surface, simplifying evaporator fabrication [86]. The limitations of some pure polymers include relatively narrower absorption bands compared to carbon and potential long-term photodegradation. Current advancements focus on creating copolymer blends for broader absorption, compositing them with other materials to enhance stability and conductivity, and exploiting their inherent flexibility for creating stretchable or shape-adaptive evaporation devices [87].

The most recent paradigm is the strategic development of hybrid and bio-inspired composite materials [88]. This approach intentionally combines two or more of the above material classes to create synergies that overcome individual limitations. Examples include decorating CNT sponges with plasmonic nanoparticles to enhance low-wavelength absorption, incorporating semiconductor nanocrystals into polymeric hydrogels to combine high heat generation with superior water management, or fabricating mineralized biopolymer composites inspired by natural structures for exceptional strength and diffusion pathways [89]. The design logic is systematic: one component may serve as the primary photothermal converter, another as a hydrophilic water transporter, and a third as a mechanically reinforcing or functionally active scaffold. This trend underscores the field’s maturity, moving from single-component materials to engineered composites where the final properties are greater than the sum of their parts [90].

In summary, the journey from classical to emerging photothermal materials reflects a strategic shift from exploiting innate properties to deliberately designing material systems with coupled optical, thermal, hydraulic, and mechanical functionalities. This progression sets the stage for the advanced material engineering strategies discussed next, where micro/nanoscale structuring and surface chemistry are leveraged to unlock unprecedented control over the evaporation process.

### 3.3. Material Engineering Strategies for Overcoming Performance Bottlenecks

**Figure 2:**
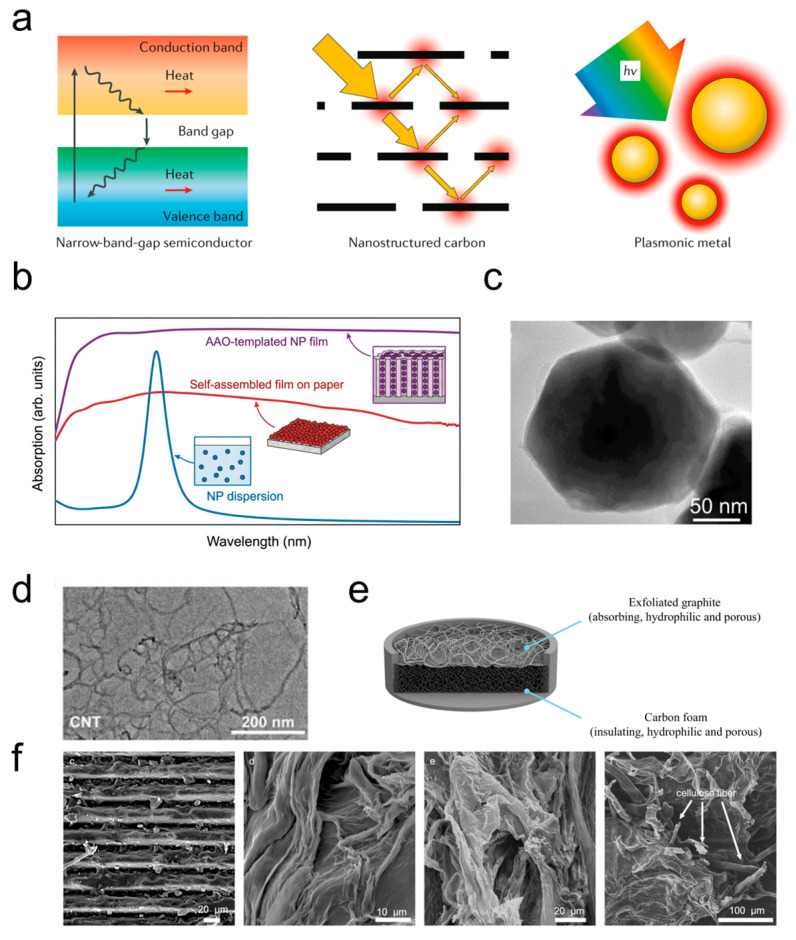
(**a**) Schematic illustration of photothermal heating mechanisms in narrow-band-gap semiconductors, carbon nanostructures, and plasmonic absorbers. Reprinted with permission from Ref. [104]. Copyright 2020 Springer Nature. (**b**) Absorption spectra of various plasmonic-based solar absorbers: NP dispersion, paper-supported self-assembled NP film, and AAO-templated NP film, with corresponding structural schematics shown in the insets [105]. (**c**) Transmission electron microscopy (TEM) images of Fe_3_O_4_@G nanoparticle at different magnification. Reprinted from Ref. [106]. (**d**) CNT composite structure. Reprinted with permission from Ref. [107]. Copyright © 2025 Wiley-VCH. (**e**) carbon foam, and (**f**) rGO-based architecture. Reprinted from Ref. [108].

While the discovery of novel photothermal substances provides the raw ingredients, the true advancement of SIE technology lies in sophisticated material engineering [91]. The path toward high-efficiency, stable, and multifunctional systems is paved by deliberate strategies that transcend intrinsic material properties (Figure 2a). These strategies address core bottlenecks such as incomplete light harvesting, inefficient water-vapor dynamics, material fragility, and functional singularity through precise design at the micro- and nanoscale. This section details four pivotal engineering avenues: spectral manipulation, surface engineering, hybridization, and functional integration, which collectively transform passive absorbers into active, high-performance components for next-generation evaporators [92].

Spectral manipulation aims to maximize the harvesting of solar energy, particularly in the near-infrared (NIR) region, which constitutes a major portion of the solar spectrum (Figure 2b,c). Moving beyond inherently broadband absorbers like carbon black, advanced micro/nanostructural designs are employed to achieve near-perfect, omnidirectional light absorption [93]. Key approaches include: (1) plasmonic nanostructuring, where arrays of metallic nanoparticles or nanopores are designed to support multiple resonant modes and magnetic polaritons, creating a graded refractive index that traps light; (2) photonic structures and metamaterials, which utilize coherent interference in dielectric multilayers or precisely patterned meta-surfaces to engineer absorption peaks and create “anti-reflection” effects; and (3) hierarchical porous networks, commonly found in aerogels or foams, which promote multiple internal scattering of light, dramatically increasing the optical path length, and ensuring that photons are absorbed within the material (Figure 2d–f). For instance, constructing a vertically aligned graphene nanosheet array or a silicon nanowire forest can achieve absorptance exceeding 99% across the solar spectrum by effectively capturing light over a wide range of angles and wavelengths [94].

Surface engineering focuses on the critical liquid–solid–gas interface by precisely tuning surface wettability [41]. The goal is to establish a dynamic balance between rapid capillary water supply to the heating zone and unobstructed vapor escape [95]. This is typically achieved by constructing surfaces with controlled hydrophilicity/hydrophobicity gradients. An ideal evaporator surface often features micro/nanoscale roughness combined with chemical modifications to create a super-hydrophilic state, ensuring continuous water film formation for efficient thermal contact. More advanced designs involve Janus (asymmetric) wettability, where the top photothermal layer is moderately hydrophilic or even hydrophobic to facilitate vapor nucleation and release, while the underlying water transport layer is highly hydrophilic to pump water upward against gravity and thermal gradients. This directional water management, inspired by natural systems like tree trunks, is fundamental for preventing salt accumulation at the evaporation front and maintaining sustained high performance [96].

Hybridization and composition represent a cornerstone strategy for creating materials with synergistic properties that no single component can possess [51]. The logic is to combine materials to compensate for each other’s weaknesses and amplify their strengths. Common paradigms include: (1) elastic matrix reinforcement, where brittle but highly absorptive materials like graphene oxide or MXenes are incorporated into a flexible polymer (e.g., polyvinyl alcohol, bacterial cellulose) or hydrogel network, yielding a composite that is simultaneously mechanically robust, thermally conductive, and photothermally active; (2) plasmonic enhancement, where plasmonic nanoparticles are anchored on semiconductor or carbon substrates to inject “hot electrons” or enhance local fields, boosting low-wavelength absorption; and (3) multi-scale porous structuring, where different materials form interconnected primary (nano) and secondary (micro) pores, optimizing pathways for light, water, and vapor. The interface between components is crucial; strong chemical or physical bonding ensures efficient heat transfer and structural integrity during long-term operation and hydration cycles [97].

Functional integration elevates the role of the photothermal material from a mere heat generator to a multi-task platform [98]. By incorporating specific active components, the evaporator can perform valuable auxiliary functions alongside water production: (1) self-cleaning and anti-biofouling can be achieved by integrating photocatalytic nanomaterials that generate reactive oxygen species under light to degrade organic contaminants and microbes, or by creating super-hydrophobic surfaces that prevent biofilm adhesion; (2) in situ pollutant degradation involves using the photothermal heat and/or photoexcited carriers to drive Fenton-like reactions or accelerate the breakdown of dyes, pharmaceuticals, or heavy metal complexes; (3) energy harvesting is realized by coupling the evaporator with mechanisms for converting waste energy into electricity. Examples include incorporating thermoelectric modules below the evaporator to utilize the temperature gradient or using flowing saline water over charged nanochannels within the material to generate osmotic or streaming potentials. This transforms a passive distillation unit into an active, multi-output system for concurrent water–energy–resource recovery [99]. Table 3 summarizes the objectives, representative methods, and outcomes of these core engineering strategies.

A critical assessment of scalability is essential to distinguish material and structural classes with genuine commercial potential from those that are unlikely to be deployed [100]. The most promising candidates for real-world application include biomass-derived carbons (e.g., carbonized wood, coconut char, biochar) and conductive polymers (e.g., polypyrrole, PEDOT:PSS), which can be produced at low cost (<$1–10 per m^2^) using established industrial processes (pyrolysis, roll-to-roll coating, or in situ polymerization) while offering adequate mechanical flexibility and environmental stability [101]. In contrast, several classes are currently not scalable: noble-metal plasmonic materials (Au, Ag) are economically prohibitive due to high cost and potential ion leaching; complex 3D architectures (e.g., precisely engineered fin arrays or multi-material photonic structures) rely on low-throughput, high-cost methods such as lithography, electrospinning, or multi-step templating; and many laboratory-scale hydrogel systems suffer from poor mechanical strength, swelling-induced deformation, and limited long-term outdoor stability [102]. MXenes and COFs exhibit excellent photothermal performance but currently require hazardous or expensive synthesis (HF etching, solvothermal conditions, costly monomers); their scalability awaits future breakthroughs in green chemistry [103]. Therefore, future research should prioritize low-cost, scalable materials and structures, and mandatory reporting of cost-per-square-meter estimates and long-term durability tests should be required to objectively assess practical viability. In conclusion, material engineering is the linchpin that unlocks the full potential of photothermal substances for practical SIE. By intelligently manipulating light–matter interactions, fluidic interfaces, material composites, and multifunctional elements, researchers can systematically overcome the inherent limitations of raw materials. These engineered constructs no longer merely absorb light; they actively manage the entire energy and mass transfer process, setting a new standard for performance and opening the door to complex, adaptive, and resilient solar-powered water production systems.

## 4. Evaporator Structural Design: Synergistic Management of Water, Heat, and Salt

The journey from a high-performance photothermal material to an efficient and robust solar evaporator necessitates deliberate structural design. While the material defines *what* absorbs light, the structure dictates *how* water is supplied, heat is localized, vapor escapes, and salt is managed. This section focuses on the architecture of the evaporator itself, examining how spatial and component design can actively orchestrate the critical yet often competing fluxes of water, heat, and salt ions to achieve sustained high performance. We begin by deconstructing the fundamental building blocks of a typical evaporator.

### 4.1. Fundamental Structural Components and Their Roles: From Functional Decoupling to Integrated Design

**Figure 3:**
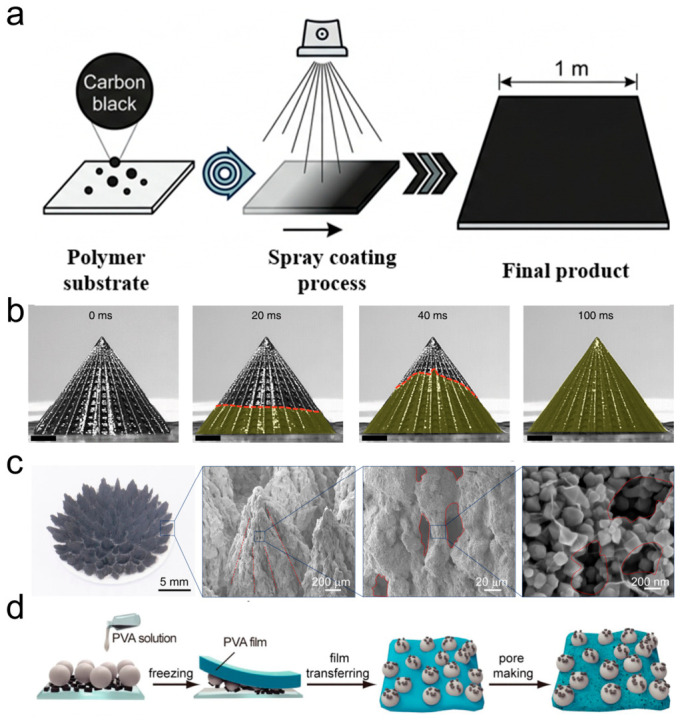
Schematic illustrations of different types of solar interfacial evaporators. (**a**) Planar evaporator design. (**b**,**c**) Three-dimensional (3D) evaporator configurations. Reprinted from Refs. [106,128] with permission. (**d**) Janus evaporator featuring asymmetric wettability or structural design. Reprinted with permission from Ref. [129]. Copyright © 2023 Wiley-VCH.

The architecture of most solar interfacial evaporators can be conceptually decomposed into three functionally specialized layers: the photothermal layer, the water transport layer, and the thermal insulation layer (Figure 3a). This functional decoupling represents the foundational design philosophy, where each layer is optimized for a primary physical role, simplifying material selection and initial analysis [37].

The photothermal layer is the solar energy harvesting and interfacial heating engine [109]. Positioned at the top, its primary functions are to maximize broadband solar absorption and convert photons into heat with high efficiency at the water–air interface. The key metrics for this layer are solar absorptance and in-plane thermal conductivity. A high in-plane thermal conductivity helps distribute heat laterally, preventing localized hot spots that can lead to premature water film dry-out and salt crystallization. Materials range from deposited nanoparticle coatings and carbon-based fabrics to porous semiconductor films [110]. Beneath it, the water transport layer acts as the hydraulic supply network. Its core function is to provide a continuous, low-resistance capillary pathway to deliver bulk water to the overheated photothermal layer, compensating for the loss due to evaporation. Its performance is governed by porosity, pore size distribution, and surface hydrophilicity. An ideal water transport layer exhibits a hierarchical porous structure: micropores or nanoscale channels generate strong capillary forces, while interconnected macropores reduce flow resistance, ensuring sufficient water flux even under high evaporation rates [111]. Natural wood, cotton fabrics, aerogels, and foam ceramics are common choices.

The thermal insulation layer, typically at the bottom, is crucial for the principle of interfacial heat localization. Its function is to minimize conductive heat loss from the photothermal layer to the bulk water body underneath, thereby confining thermal energy at the evaporation interface [112]. This is achieved by using materials with very low thermal conductivity (e.g., polystyrene foam, polyurethane, air-laid paper) or by incorporating an air gap. Effective insulation dramatically increases the solar-to-vapor conversion efficiency by ensuring that most of the generated heat is utilized for phase change rather than heating the reservoir. However, this decoupled, multi-layer stack approach has inherent limitations for advanced applications. (1) Thermal contact resistance: The interfaces between different material layers can introduce significant thermal contact resistance, impeding heat transfer from the photothermal layer to the water supply and creating undesirable thermal bottlenecks. (2) Mechanical integrity and delamination: The differential swelling, thermal expansion, or long-term hydration cycles of dissimilar materials can lead to layer separation, degrading performance and durability. (3) Complexity in scalability and fabrication: Assembling and bonding multiple discrete layers complicates large-scale, low-cost manufacturing [113].

To overcome these challenges, the field has evolved toward integrated and monolithic design. This paradigm seeks to merge or blur the boundaries between the traditional functional layers, creating a unified structure where a single, rationally designed component performs multiple roles synergistically, for example, using a single material that possesses both excellent photothermal properties and inherent hydrophilicity/porosity. A prime example is naturally derived wood. Its cellulose microchannels provide superb capillary water transport, while carbonizing its surface creates a native, integrated photothermal layer. The bulk wood itself serves as a partial thermal insulator due to its low cross-plane thermal conductivity [114].

Instead of stacking discrete layers, a single porous monolith (e.g., a graphene oxide hydrogel, a ceramic foam) is engineered with structural gradients [115]. For instance, the top surface can be densified or coated to enhance light absorption, while the bulk and bottom maintain an open, hydrophilic porous network for water pumping and thermal insulation. The gradient is continuous, eliminating sharp interfaces [116]. Growing photothermal nanomaterials (e.g., MoS_2_ nanosheets, polypyrrole) directly onto a 3D porous scaffold [117] creates a strong chemical/physical bond, integrating the photothermal function into the skeleton of the water transport and insulating matrix, resulting in a cohesive, robust device. This transition from a stack of functional layers to a functionally graded monolith represents a significant leap in evaporator design. It reduces internal resistances, enhances mechanical and operational stability, and simplifies fabrication pathways. This integrated philosophy sets the stage for the more advanced structural morphologies—such as 2D, 3D, and Janus designs—discussed in the following sections, which further exploit geometry to master the synergy between water, heat, and salt [118].

### 4.2. From 2D to 3D: Structural Evolution and Performance Leap

The evolution of evaporator architecture from simple two-dimensional (2D) configurations to sophisticated three-dimensional (3D) and Janus structures represents a paradigm shift in performance and functionality (Figure 3b,c). This progression is driven by the need to overcome the fundamental limitations of early designs and to actively harness, rather than merely mitigate, environmental factors. Each structural paradigm embodies distinct principles for managing the interplay of light, heat, water, and salt [119].

Two-dimensional (2D) floating structures represent the most foundational design. In this configuration, a flat photothermal sheet or fabric floats directly on the water surface, relying on passive capillary wetting for water supply [120]. Their primary strengths lie in simplicity, low material consumption, and ease of fabrication, making them ideal platforms for screening novel photothermal materials and studying interfacial phenomena [51]. Critically, however, their performance is fundamentally bounded: the evaporative area is limited to the geometric projection, capping vapor generation per footprint [121]. Moreover, 2D designs are highly susceptible to environmental losses—heat dissipation to bulk water, convective loss to ambient air, and rapid salt fouling on the evaporative surface. These limitations are not merely incremental but structural; without sophisticated insulation or active salt management, 2D systems are impractical for real-world desalination or prolonged outdoor operation. Their angular sensitivity to sunlight further constrains daily energy yield unless coupled with solar tracking, which defeats their cost advantage [122].

Three-dimensional (3D) volumetric structures overcome several of these inherent ceilings by extending the evaporator into both air and water, creating spatially expanded domains for evaporation and energy exchange [123]. The key advancement is decoupling evaporative area from footprint, enabling evaporation rates that can exceed the theoretical 2D limit under one-sun illumination. More importantly, 3D designs convert previously detrimental environmental factors into gains: cooler sidewalls harvest ambient thermal energy via convection and conduction, effectively recycling energy [124]. However, this performance leap comes at significant costs. First, 3D geometries inevitably increase material usage and fabrication complexity (e.g., molding, templating, or 3D printing). Second, their volumetric nature can introduce structural fragility, particularly for porous monoliths under mechanical stress. Third, while salt transport is often improved compared to 2D designs, many 3D structures still suffer from salt accumulation at evaporation fronts unless carefully engineered with graded porosity or specific surface wettability. Thus, the choice of 3D design must balance flux enhancement against durability and manufacturing feasibility [125].

Janus (asymmetrical) structures introduce a directional design philosophy, most commonly featuring a hydrophobic top layer and a super-hydrophilic bottom substrate (Figure 3d). This asymmetry enables active fluid management: the hydrophilic bottom pumps water upward, while the hydrophobic top confines menisci to underlying pores, creating a hydrostatic pressure gradient that drives salt ions away from the evaporation front [126]. The critical advantage is passive, directional salt rejection, enabling long-term stability in hypersaline brines without external cleaning. However, Janus designs are not without drawbacks. Precise control of bilayer interface properties (thickness, porosity, adhesion) is essential yet challenging, often requiring specialized fabrication methods such as electrospinning, spray coating, or plasma treatment. An overly hydrophobic top layer can reduce vapor escape rates, paradoxically lowering evaporation flux despite excellent salt rejection. Furthermore, many Janus structures sacrifice the ambient energy-harvesting capability typical of 3D designs because their sidewalls are often not optimized for convective heat gain. Consequently, Janus configurations excel in high-salinity desalination where stability is paramount, but may be suboptimal for maximizing evaporation rate under low-salinity or freshwater conditions [127].

Table 4 summarizes the key characteristics of these three structural paradigms. In practical terms, no single design dominates all performance metrics. 2D structures remain valuable for fundamental research and low-cost, short-term applications where salt accumulation is manageable. 3D volumetric designs are preferred when maximizing vapor generation per footprint under varying solar angles, provided that fabrication complexity and material robustness are acceptable. Janus architectures are indispensable for the long-term desalination of high-salinity brines (e.g., reverse osmosis concentrate or produced water), where passive salt rejection outweighs moderate reductions in evaporation flux. Future advances will likely involve hybrid designs—such as 3D Janus or gradient-wettability 3D structures—that combine ambient energy harvesting with directional salt management, moving beyond simple structural descriptors toward function-integrated systems.

### 4.3. Salt Rejection and Anti-Fouling Strategies

**Figure 4:**
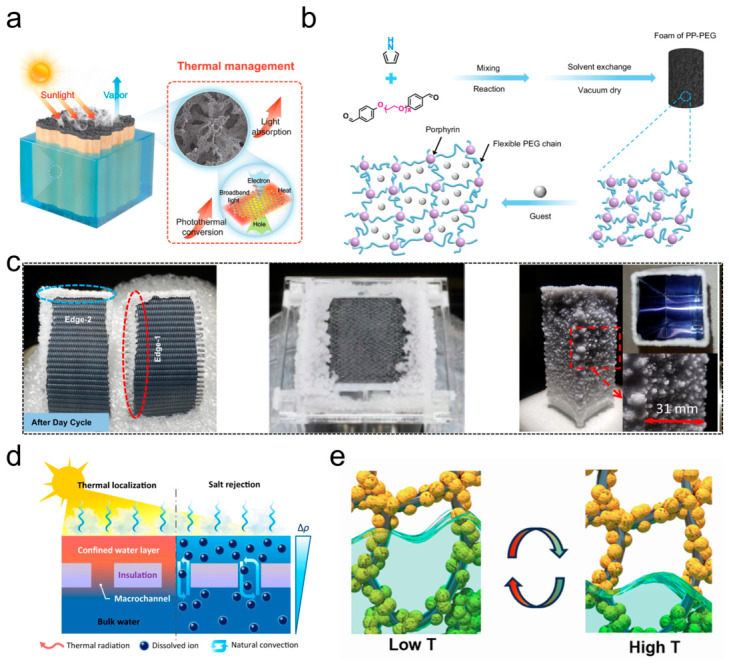
Advanced salt-resistant evaporator designs. (**a**) Schematic diagram of lignin-engineered reconstituted woody framework strategy. Reprinted from Ref. [147]. (**b**) Design of PP-PEG 3D foams prepared by the reaction of pyrrole with dialdehyde precursors containing PEG chains, and its adaptability to the guest. Reprinted from Ref. [148]. (**c**) Edge-crystallization evaporator with salt-resistant design. Reprinted from Refs. [141,149,150]. (**d**) Simultaneous thermal localization and salt rejection via a wick-free confined water layer structure. Reproduced from Ref. [140] with permission. (**e**) Bilayer solar evaporator with thermo-responsive self-regulating function. Adapted from Ref. [92].

Sustained high performance in real-world solar desalination and wastewater treatment is fundamentally challenged by two interrelated phenomena: salt crystallization and surface fouling [130]. These processes can block water pathways, reduce light absorption, and degrade materials, leading to irreversible performance decline. Consequently, developing robust strategies to manage salt and prevent fouling is not merely an enhancement but a critical requirement for practical application. Current approaches have evolved from simple passive resistance to active and even adaptive management, forming a multi-layered defense system for evaporator durability [131] (Figure 4). However, a critical examination reveals that no single strategy is universally superior; each involves inherent trade-offs between energy consumption, structural complexity, long-term reliability, and operational range. The choice of strategy must be guided by specific water chemistry and operating conditions rather than by peak performance metrics alone.

Passive repulsion strategies aim to prevent the adhesion of scaling ions or foulants at the evaporative interface through inherent material or surface properties [132] (Figure 4a,b). A primary focus is on engineering surface wettability. Creating a stable water layer on a super-hydrophilic surface can form a physical barrier against salt crystals and oil droplets [133]. More advanced designs employ underwater super-oleophobic surfaces, combining micro/nanostructures with hydrophilic chemistry to trap water and form a repulsive hydrated layer, effective for oil-contaminated water. Another passive approach uses ion-rejection membranes or charged hydrogels containing fixed charged groups that electrostatically repel similarly charged ions, slowing salt accumulation [134]. The key strength of passive strategies is their energy-free operation and material-level simplicity. [135]. Critically, however, their efficacy is fundamentally limited by capacity. Without a mechanism to remove rejected ions or foulants from the vicinity, local concentration builds inexorably. Under high salinity (e.g., >10 wt% NaCl) or prolonged operation, passive repulsion eventually saturates and fails, often abruptly. Moreover, super-hydrophilic surfaces can become compromised by organic fouling or biofouling, which alters the surface chemistry. Thus, passive strategies are best suited for low-to-moderate salinity feeds (e.g., brackish water, seawater) and short-term operation, but are inadequate for hypersaline brines or long-term continuous desalination.

Active expulsion strategies acknowledge that complete prevention is often impractical; therefore, they focus on controlling where and how salt crystallizes, facilitating its continuous removal from the system [136]. The dominant paradigm is directed salt crystallization (Figure 4c). By designing structures with localized evaporation fronts and maintaining other areas wetter, salt ions are preferentially transported to and crystallize at these designated “sacrificial” sites, keeping the primary evaporative surface clean [137]. This is often synergized with gravity-assisted salt draining in 3D structures, where concentrated brine, being denser, naturally flows downward, carrying salt away from the evaporation zone [138] (Figure 4d). A more dynamic active strategy is dissolution–diffusion recirculation. In this design, periodically or continuously introducing a small amount of fresh or low-salinity water from the sides or bottom can dissolve surface salt crystals and create a convective flow that carries ions back into the bulk solution, establishing a sustainable salt balance. However, these advantages come at a cost. Directed crystallization requires the precise control of wettability gradients and geometry; poor design can lead to salt bridging or the blockage of sacrificial sites. Gravity-assisted draining works only in certain 3D configurations and loses effectiveness in microgravity or inclined orientations. Dissolution–diffusion recirculation consumes additional water and may dilute the produced vapor or require external flow control. Furthermore, active strategies typically increase fabrication complexity and material usage. Therefore, active expulsion is the method of choice for hypersaline brine treatment (e.g., reverse osmosis concentrate, produced water) where passive methods fail, but may be over-engineered for low-salinity applications where simpler 2D or passive designs suffice [139].

Recent mechanistic studies have elucidated that the interplay between convective and diffusive ion transport governs salt crystallization patterns in porous solar evaporators. In conventional wick-fed designs, capillary-driven flow dominates, producing non-selective ion advection that results in mixed salt deposition across the evaporator surface [140,141]. In contrast, when the pore size is reduced below a critical threshold (e.g., <0.1 μm) and the diffusion length is optimized, convection is effectively suppressed, rendering ion transport diffusion-controlled. Under this regime, the disparity in ion diffusion coefficients, which enables selective back-diffusion of the more mobile ions into the bulk solution, while the less mobile ions accumulate at the evaporation front and crystallize preferentially. This principle, termed diffusion-driven selective crystallization (DiSC), has been validated by COMSOL Multiphysics 6.1 and confirmed experimentally, achieving >99% purity of NaCl directly from mixed brines or natural seawater without any pre- or post-treatment [142]. The strategy has been successfully extended to other ion pairs (Ba^2+^/K^+^, M^2+^/Li^+^), underscoring the universal role of diffusion coefficient differences in dictating selective crystallization.

An orthogonal yet complementary mechanism involves the coupling of thermal and solutal Marangoni effects, which arise from surface tension gradients along the gas–liquid interface. In three-dimensional evaporators featuring asymmetric grooves or conical profiles, spontaneous water film formation leads to axial gradients in film thickness and temperature: the apex, characterized by a thinner film and enhanced evaporative cooling, exhibits a lower temperature than the base [128]. This temperature disparity generates a surface tension gradient that drives thermocapillary flow from the warmer, thicker bottom region toward the cooler apex, a phenomenon described by τ=(dγ/dT)·(ΔT/L) [143]. Such flow not only sustains water supply to the most active evaporation sites but also directionally transports salt ions, culminating in localized crystallization exclusively at the apex or peripheral edges, thereby preserving the optical and thermal functionality of the photothermal surface. Infrared thermography and micro-computed tomography have confirmed that this spatially confined crystallization does not degrade evaporation efficiency, and the accumulated salt can be readily detached. Furthermore, in confined water layer architectures with macrochannels of optimized diameter (e.g., 2.5 mm), salinity-gradient-driven natural convection accelerates salt backflow while incurring negligible additional heat loss, enabling simultaneous thermal localization and effective salt rejection even at 20 wt% NaCl [140]. Collectively, these mechanistic insights—grounded in numerical modeling, in situ flow visualization, and quantitative ion analysis—transform salt management from an empirical challenge into a rationally engineered process governed by fundamental transport phenomena.

Dynamic and adaptive regulation represents the frontier of smart evaporator design (Figure 4e). This strategy employs stimuli-responsive materials that can change their surface properties (e.g., wettability, ionic state) in situ in response to environmental triggers, allowing the evaporator to self-adjust its anti-fouling mode [144]. Common triggers include integrating photothermal materials with photo-switchable molecules that can make a surface switch between hydrophobic and hydrophilic states upon light irradiation, potentially disrupting early-stage biofilm attachment or altering water meniscus shape [40]; utilizing thermo-responsive polymers like poly (N-isopropylacrylamide) (PNIPAM), which undergo a hydrophilic-to-hydrophobic transition above a lower critical solution temperature (LCST) [145] so the evaporator’s own photothermal heat could trigger this switch, potentially creating a “self-cleaning” hydrophobic state to shed aggregates; and employing pH-responsive polymers or surfaces that change charge or wettability with the local pH, which might be altered by salt concentration or microbial activity. While highly promising for creating adaptive, self-regulating systems, dynamic strategies are currently in earlier stages of development, facing challenges related to the long-term cycling stability of responsive materials. First, the cycling stability of responsive materials is often poor; after tens or hundreds of switches, performance degrades due to molecular fatigue or leaching. Second, integration into macroscopic, robust evaporators remains difficult—most demonstrations are on thin films or small-scale devices. Third, the control precision is inherently limited: triggers such as light or temperature gradients may be non-uniform across the evaporator surface, leading to partial or delayed response. Fourth, the photothermal heat that triggers LCST switching may also accelerate salt precipitation before the switch occurs, causing a “race condition”. Thus, dynamic strategies are currently at a proof-of-concept stage, promising, but not yet field-ready for real-world desalination. Their primary value lies in revealing new mechanisms for adaptive interfaces, rather than offering an immediately practical solution [146].

The mechanisms, representative implementations, and key considerations associated with these three tiers of strategies are given in Table 5.

### 4.4. Thermal Energy Management Strategies

The pursuit of ultra-high solar-to-vapor efficiency in interfacial evaporation has catalyzed the evolution of thermal management from a peripheral consideration to a central design pillar [151]. Sophisticated strategies now focus not only on minimizing inevitable losses, but also on actively recovering waste heat and synergistically coupling with environmental energy flows (Table 6). This holistic approach transforms the evaporator from an isolated energy converter into an integrated component within a broader thermal ecosystem, pushing efficiency toward and beyond the theoretical single-stage limits [152].

**Figure 5:**
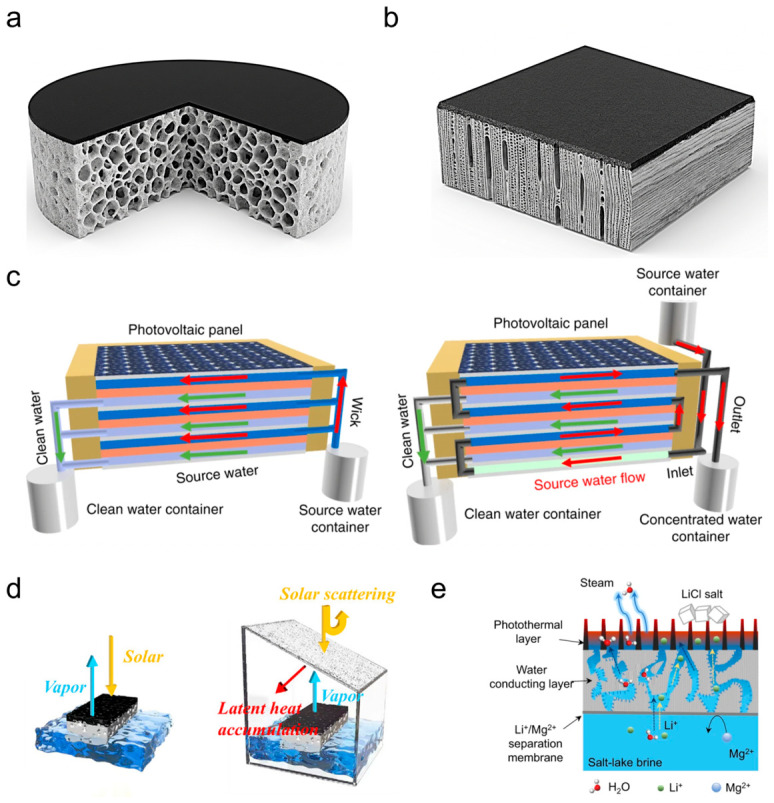
Advanced functional evaporator designs. (**a**) Aerogel-based evaporator. (**b**) Wood-derived evaporator. (**c**) Schematic illustration of the integrated photovoltaics-membrane distillation (PV-MD) devices. Reprinted from Ref. [160]. (**d**) Radiative cooling-assisted solar evaporator. Reprinted with permission from Ref. [161]. Copyright © 2026 Wiley-VCH. (**e**) Polyamide membrane-based solar-driven lithium extraction. Reprinted from Ref. [162].

Minimizing thermal loss is the foundational strategy, targeting the three classical pathways of heat dissipation. Reducing conductive loss is primarily achieved through the use of low-thermal-conductivity substrates, such as polystyrene foam, wood, or aerogels, which isolate the photothermal layer from the underlying bulk water [153] (Figure 5a,b). To combat radiative loss, engineered surfaces with low mid-infrared emissivity, often leveraging reflective metal layers or specialized photonic structures, are employed to suppress blackbody radiation from the hot surface to the cooler sky. Finally, suppressing convective loss involves structural designs that create a stagnant air layer or micro-enclosure above the evaporative interface, effectively shielding it from wind-induced cooling [154]. A prime example is the use of transparent, porous insulating covers or the design of confined evaporation channels within monolithic aerogels, which can collectively reduce the total parasitic heat loss to below 10% of the absorbed energy.

Moving beyond loss prevention, advanced systems incorporate heat recovery and reutilization to elevate the overall energy budget. The most direct method is condensate heat recovery, where the latent heat released during vapor condensation is captured [155]. This is ingeniously implemented in cascaded multi-stage or multi-layer systems (Figure 5c). In a typical two-stage design, the condensing surface of the first stage acts as the heating source for the second, evaporating additional water at a lower temperature [156]. This recycling of latent heat can boost the total daily water yield per unit solar input by 50% or more compared to a single-stage device. Furthermore, internal heat recycling within a single evaporator, such as preheating incoming feedwater with the outgoing brine in a counter-flow design, further enhances the sensible heat utilization [157].

The most innovative frontier is environmental energy coupling, which aims to break the conventional system boundary by harnessing additional, non-solar energy fluxes [158] (Figure 5d,e). A powerful paradigm is the integration of radiative cooling with solar heating. Here, the top surface of the evaporator is designed to be a spectral-selective material that strongly absorbs sunlight while simultaneously emitting mid-infrared radiation through the atmospheric transparent window (8–13 μm) to the cold universe (≈3 K) [159]. This creates a passive cooling power that can lower the condenser temperature below the ambient, significantly increasing the vapor condensation rate and driving force without any extra energy input. Conversely, systems can be designed to utilize low-grade waste heat as a supplementary or primary thermal source, enabling continuous 24/7 operation and dramatically improving land and resource utilization efficiency [37].

**Table 6:** Quantitative comparison of key performance metrics for representative SIE systems.

	Evaporator Type	Evaporation Rate (kg·m^−2^·h^−1^)	Solar-to-Vapor Efficiency (%)	Stability Duration	Salt Tolerance	Ref.
1	Au/nanoporous alumina plasmonic absorber	–	–	–	–	[163]
2	MnO_2_ decorated film	2.24	81.70%	–	–	[164]
3	Graphene foam	–	~91.4%	–	–	[165]
4	MOF-derived carbon/MXene	–	~93.4%	–	Good	[166]
5	3D cup-shaped absorber	–	~100%	–	–	[167]
6	PPy coated 3D conical	1.7	93.80%	–	–	[168]
7	V-shaped evaporator	1.6	–	–	–	[169]
8	Janus CB@PMMA/PAN	–	–	16 days (3.5 wt% NaCl)	Excellent	[170]
9	CNTs-PPy/hydrophilic CNTs-PPy)	–	–	150 min salt removal	Good	[171]
10	Janus carbonized chitosan aerogel	–	–	180 min (20 wt% brine)	Good	[172]
11	Janus (graphene/PDMS)	1.38	–	–	Self-recovering hydrophobicity	[173]
12	hydrophilic/hydrophobic Janus	1.60	–	30 days (3.5 wt% NaCl)	Excellent	[174]
13	CNTs@SiO_2_ nanofibrous aerogel	~1.5	–	Long-term (0–20 wt% brine)	Excellent	[175]
14	Water hyacinth-inspired foam	2.09	–	–	Good	[176]
15	Sorghum stalk derived honeycomb evaporator	3.79	–	–	Good	[177]
16	Structurally graded aerogel	1.94	–	8 h stable	Good	[178]
17	Nanofibrous hydrogel	2.85	–	–	Good	[179]
18	3D-printed hydrogel	–	–	–	Good	[24]
19	Hydrogel evaporator with vertical radiant vessels	3.53	–	–	Excellent	[180]
20	Polyzwitterionic hydrogel	3.70	–	–	Excellent	[181]
21	Ion-selective Janus hydrogel	6.86	–	–	Excellent	[182]
22	Porphyrin-based polyCOF foam	4.89	–	–	–	[148]
23	MGH hydrogel	3	90%	–	–	[183]
24	Bamboo fiber	1.79	94.40%	1800 h	Excellent	[184]
25	V-shaped evaporator with radiative cooling	2.06	–	–	–	[185]

## 5. System Integration and Energy–Water Coproduction

**Figure 6:**
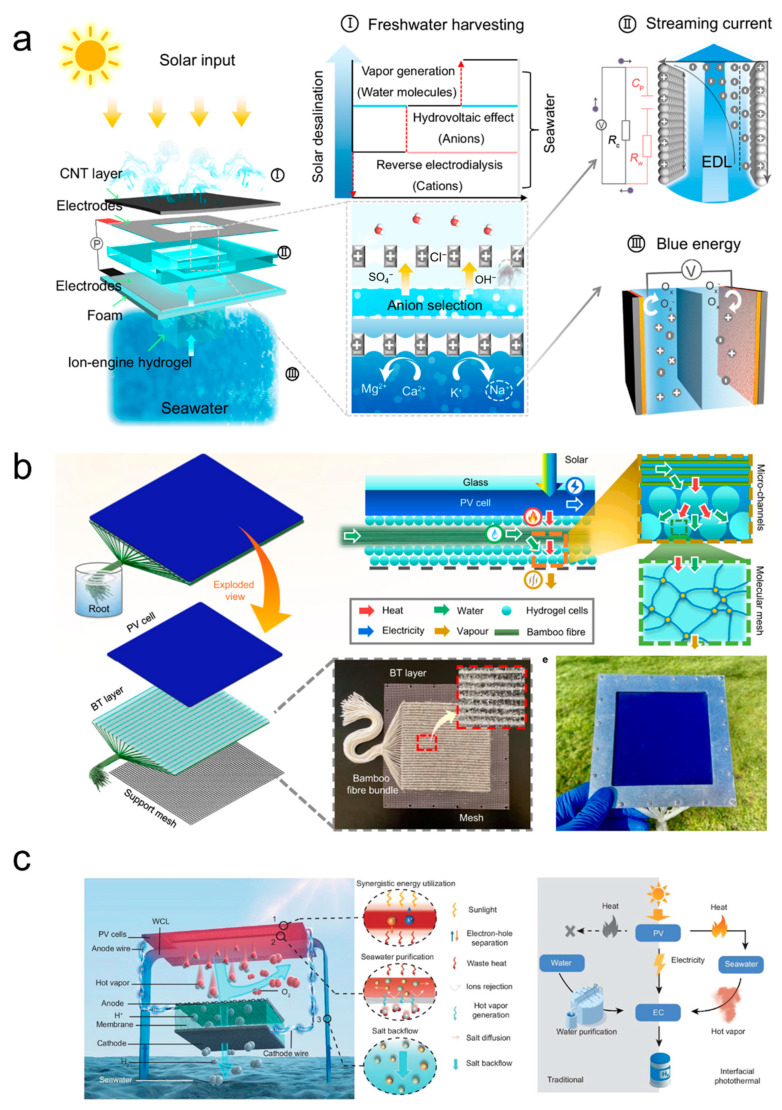
Integrated systems for solar-driven water-energy nexus applications. (**a**) Solar interfacial water-electricity cogeneration device. Reprinted from Ref. [204]. (**b**) PV leaf-inspired water-electricity cogeneration device. Reprinted from Ref. [203]. (**c**) Interfacial solar steam electrolyzer for hydrogen generation. Reprinted from Ref. [202].

The evolution of solar-driven interfacial evaporation (SIE) is undergoing a pivotal paradigm shift: from optimizing standalone evaporators for a single output (water) toward engineering integrated systems for multifunctional coproduction. This shift recognizes the evaporative interface not merely as a site for water phase change, but as a versatile thermal and chemical platform capable of synergistically managing energy and mass flows. By strategically coupling the evaporation process with power generation cycles or resource recovery mechanisms, these integrated systems significantly enhance overall energy utilization, economic viability, and application scope, moving decisively beyond the limitations of traditional solar stills (Figure 6).

### 5.1. From Pure Evaporation to Multifunctional Systems

The conventional solar still represents a linear, single-function technology. Modern research breaks this mold by designing systems where freshwater production actively enables or is accompanied by the generation of electricity or the extraction of valuable resources, creating circular and synergistic processes [94,186]. Water–energy coproduction systems capitalize on various forms of dissipated energy during evaporation, converting them into electricity (Figure 6a). This creates a dual-output system from a single solar input. This approach exploits the inherent temperature gradient between the hot evaporative surface and the cooler bulk water. Integrating flexible TE modules (e.g., based on Bi_2_Te_3_) beneath the evaporator allows for direct conversion of this thermal difference into electricity [187]. The key challenge lies in optimizing the thermal contact resistance and matching the TE material’s properties with the evaporator’s operating temperature.

Particularly relevant for desalination, this method harvests the osmotic energy created between the concentrated brine near the evaporator and the bulk seawater. By integrating ion-selective membranes into the evaporator structure, a continuous ion flux can be driven, generating electricity [111]. A notable study detailed a design where the water evaporation process spontaneously maintained the salinity difference, enabling stable and continuous osmotic power generation alongside freshwater production, showcasing a clever use of the desalination byproduct (concentrated brine) [188]. Mechanisms such as triboelectric or hydrovoltaic effects can harvest energy from the flow of vapor or water within the evaporator’s porous structure. While current power densities are modest, this principle offers a pathway to electricity generation without requiring solid-state thermal gradients [189].

The progression toward these multifunctional systems marks a significant maturation of SIE technology [190]. It elevates the technology’s value proposition by improving the overall energy efficiency (e.g., utilizing waste heat or salinity gradients) and creating additional revenue streams (e.g., from generated electricity or recovered materials), which is crucial for overcoming economic barriers to large-scale adoption (Figure 6b). The next frontier lies in intelligently managing the potential trade-offs between different functions and scaling these integrated concepts into robust, real-world applications [191].

### 5.2. Photothermal-Photocatalytic/Photoelectrochemical Coupling Systems

Building upon the paradigm of multifunctionality, a particularly sophisticated frontier in SIE research is the marriage of photothermal conversion with photocatalytic (PC) or photoelectrochemical (PEC) processes [192]. This integration moves beyond simple side-by-side co-production, aiming for a synergistic interplay at the molecular level. The core concept leverages the heat generated from photothermal conversion not merely as an energy source for evaporation, but as a critical physical catalyst to dramatically enhance the kinetics and efficiency of photocatalytic reactions such as the degradation of persistent organic pollutants or the challenging water-splitting reaction for hydrogen production [193]. This creates a tightly coupled system where light, heat, and catalytic active sites work in concert to drive complex chemical transformations alongside water purification or fuel synthesis (Figure 6c).

The synergy operates through several key mechanisms where thermal energy acts as a performance multiplier for photocatalysis. According to the Arrhenius equation, increasing temperature exponentially accelerates chemical reaction rates. The localized heat at the photothermal–catalytic interface directly boosts the rate of surface redox reactions involved in pollutant degradation or hydrogen evolution. Heat facilitates the dissociation of photogenerated excitons (electron-hole pairs) and reduces charge recombination by increasing carrier mobility. This leads to a greater population of long-lived, reactive electrons and holes available for catalytic processes [194]. For instance, studies on systems coupling plasmonic nanoparticles with semiconductors have shown that the photothermal effect can induce a localized “hot spot” that promotes interfacial electron injection and reduces recombination losses [195]. The thermal energy can effectively narrow the bandgap of certain semiconductors, enabling them to absorb a broader spectrum of sunlight. Furthermore, in PEC water splitting, heat can significantly lower the thermodynamic and kinetic overpotentials required for the oxygen and hydrogen evolution reactions (OER/HER), making the process more energy-efficient [196].

Two primary application pathways demonstrate this powerful coupling. Here, the system is designed to simultaneously produce clean water vapor and completely mineralize dissolved organic contaminants [197]. A typical architecture involves a composite material where photothermal substrates are intimately integrated with photocatalytic nanomaterials (e.g., TiO_2_, BiOBr, g-C_3_N_4_, or MOFs) [198]. The photothermal component ensures efficient water evaporation, while the photocatalytic component degrades organics. Critically, the heat from the photothermal component elevates the local temperature around the catalytic sites, dramatically enhancing the degradation rate constant, often by an order of magnitude compared to photocatalysis alone at ambient temperature [199]. The system design is inherently more complex, often requiring a photoelectrode or an integrated photovoltaic-electrochemical component. The photothermal effect plays a crucial role in two ways: first, by pre-heating the feedwater to reduce the electrical energy needed for electrolysis; second, by integrating a photothermal layer within the PEC cell to create a localized high-temperature environment at the catalyst surface. The system utilizes full-spectrum sunlight: the PV cell provides the voltage for electrolysis, while the photothermal component vaporizes water, and importantly, uses the waste heat from the PV cell to maintain an elevated operating temperature (~48 °C). This thermal synergy resulted in a solar-to-hydrogen (STH) efficiency exceeding 15% while concurrently producing distillate, showcasing a breakthrough in integrated solar fuel and water production [200,201].

However, practical trade-offs between water production and additional functions are often overlooked. For instance, in photothermal-catalytic coupling, the incorporation of photocatalysts can reduce light absorption efficiency by 15–20%, and the slower degradation kinetics (minutes to hours) compared to evaporation (seconds) can lead to surface fouling. In PEC systems like the interfacial solar vapor electrolyzer, balancing vapor supply and electrolysis demand is critical; any mismatch degrades the overall performance [202]. Similarly, the photovoltaic leaf (PV-leaf) can utilize seawater for evaporative cooling, achieving high overall efficiency (74.5%) while simultaneously producing clean water; however, its reliance on a continuous water source may still pose challenges in hyper-arid regions with limited access to any water [203]. In seawater evaporation-induced electricity generation (e.g., ion-engine hydrogels), high current output comes at the cost of complex material design and potential long-term instability [204]. Therefore, application-specific priorities must guide system design, and future research should adopt standardized metrics to quantify these trade-offs.

### 5.3. Large-Scale System Design and Engineering Considerations

Transitioning high-performance laboratory evaporators into deployable, large-scale systems represents the critical “valley of death” that SIE technology must cross to achieve real-world impact [205]. This leap requires a paradigm shift from pursuing peak efficiency in isolated units to systematically addressing the core engineering challenges of scalability, long-term durability, operational feasibility, and economic viability. Contemporary research is therefore evolving from functional demonstrations toward the proactive design of system-level architecture, integration strategies, and full lifecycle performance [206]. System scale-up and array design is the first fundamental step toward meaningful water output. Simply replicating a high-efficiency unit does not guarantee optimal performance at scale, as it introduces complex interdependencies in light, heat, and hydraulic distribution [207]. The key lies in modular design and array-level energy management. For instance, a modular, connectable design for all-weather water-electricity cogeneration systems has been demonstrated, providing a flexible blueprint for distributed systems. When arranging modules into arrays, optimizing spacing to prevent mutual shading and designing efficient networks for distillate collection and brine removal are essential.

A more advanced concept involves constructing thermally cascaded or hydraulically series-connected systems. A pioneering example is the “thermally boosted” multi-stage solar membrane distillation system developed by the ITEWA team. By injecting a low-grade heat source at the final stage, they significantly enhanced the vapor pressure difference across all stages, enabling an eight-stage device to achieve a water yield of 9.0 L m^−2^ h^−1^ with an exceptional overall energy efficiency of 407%, effectively overcoming the productivity decline typical in conventional multi-stage designs [1,208].

Long-term stable operation and anti-interference capability form the cornerstone of practical application. This requires translating material-level anti-salt/fouling strategies into system-level sustainable operation protocols, emphasizing “guidance” and “self-cleaning” over mere “blocking”. A notable example is a water-layer-based reverse distillation system designed to induce natural convection driven by internally generated salinity gradients, enabling stable operation in high-salinity brine (21 wt%) [209,210]. Another approach integrates intermittent maintenance into the system’s operational logic. Some designs incorporate passive or active flushing mechanisms, using feedwater during non-sunlight hours to automatically dissolve and remove salt crystals from the evaporation interface, enabling periodic regeneration without manual intervention [211].

Economic and environmental impact assessment is the ultimate metric for technology adoption [212]. Large-scale design necessitates comprehensive techno-economic analysis and life cycle assessment. This includes evaluating material costs, manufacturing energy, system lifetime, operational expenses, and the ultimate levelized cost of water. Concurrently, environmental benefits must be scrutinized from a cradle-to-grave perspective, assessing the total carbon footprint and resource consumption. A promising direction is to explore waste stream valorization, such as crystallizing and extracting salts from brine for industrial use, moving toward “zero liquid discharge” and a circular resource economy [213].

To enable a fair techno-economic assessment, we systematically collected ten representative state-of-the-art solar interfacial evaporation (SIE) prototypes from recent high-impact literature and estimated their water production costs. The estimation followed a simplified life cycle approach: material costs, laboratory-scale fabrication energy, and a conservative system lifetime of 30 days were amortized while excluding long-term maintenance, brine disposal, and post-treatment to avoid overestimating costs. Using this method, the estimated water costs for the ten advanced SIE prototypes range from approximately 5.00 to 11.00 USD·m^−3^, with the lower end achieved by high-rate, low-cost carbon/polymer systems and the higher end by devices employing expensive materials or complex multi-stage architectures. When directly compared with mature commercial desalination technologies—large-scale reverse osmosis (RO) at 0.45–0.80 USD·m^−3^ and multi-stage flash (MSF) at 0.90–2.20 USD·m^−3^—it becomes evident that even the best SIE prototypes remain one order of magnitude more expensive. This large gap persists because the SIE estimates rely on ideal laboratory conditions and short device lifetimes without auxiliary energy, whereas commercial RO and MSF benefit from decades of engineering optimization, economies of scale, and robust infrastructure. Therefore, while SIE is currently uncompetitive for grid-connected large-scale desalination, its estimated water cost (5–11 USD·m^−3^) may still be attractive for off-grid, decentralized, or emergency scenarios where diesel-driven small-scale alternatives often exceed 10–20 USD·m^−3^. The analysis underscores that future SIE research should prioritize long-term stability, system-level energy recovery, and scalable manufacturing over solely pursuing higher evaporation rates or lower material costs (Table 7).

In addition to the fundamental challenges discussed above, transitioning SIE technology from laboratory-scale prototypes to practical, commercially viable systems requires addressing critical engineering barriers related to large-scale manufacturing, production costs, and long-term operational reliability. Scalable manufacturing techniques currently under exploration include roll-to-roll (R2R) processing for flexible photothermal membranes, 3D printing for custom-designed evaporator geometries, continuous dip-coating or spray-coating for absorber layers, and template-assisted casting for hierarchical porous structures [43]. However, each method faces trade-offs between throughput, pattern precision, and material waste. For instance, while R2R enables high-speed production of 2D Janus or bilayer membranes, it struggles with uniform deposition on 3D architectures. 3D printing offers geometric freedom but suffers from low throughput and high equipment costs. Production cost analysis must account for raw material expenses, energy consumption during synthesis/fabrication, labor, and post-processing steps [214]. For most reported evaporators, the cost per square meter remains too high for competitive desalination. Biomass-derived carbons and polymer-based hydrogels currently offer the lowest cost potential, while plasmonic and MXene-based systems require significant cost reduction or recycling strategies. Engineering barriers toward commercialization include: (i) module durability under real-world fluctuating conditions (wind, waves, biofouling, UV degradation); (ii) salt management at large scale, while laboratory devices demonstrate salt resistance, scaling to square-meter or larger areas introduces non-uniform water flow, localized hot spots, and unpredictable crystallization patterns; (iii) system integration with condensation, collection, and energy storage components, ensuring minimal heat loss and high water yield over extended periods; (iv) maintenance and cleaning protocols that are low-cost and do not require system shutdown; and (v) end-of-life disposal or material recyclability to avoid secondary environmental pollution. Addressing these challenges requires close collaboration between materials scientists, process engineers, and industry partners, as well as standardized techno-economic assessment (TEA) frameworks to benchmark laboratory innovations against existing desalination technologies [215].

**Table 7:** Performance and estimated water cost of representative SIE prototypes vs. commercial desalination.

No.	Evaporator Design	Core Material/Structure	Evaporation Rate (kg·m^−2^·h^−1^)	Estimated Water Cost (USD·m^−3^)
1	3D fin structure [216]	Carbon-based foam, 3D macro-structure	7.6	5.50–7.00
2	Spatial patterned interface (SPE) [217]	Biochar, patterned heat-exchange platforms	1.68	8.00–10.00
3	8-stage condensation-evaporation system [218]	Copper foam + MOF, multistage latent heat recovery	4.64	6.50–8.50
4	PCM-integrated hydrogel evaporator [219]	Hydrogel + phase-change material (eicosane)	3.52	7.00–9.00
5	Windmill-inspired 3D evaporator (IWS) [220]	Carbon/polymer, 1–3 mm through-channels	4.95	5.00–6.50
6	Leaf-inspired 2D photothermal fabric [221]	Textile + carbon coating, porous array	2.6	6.00–8.00
7	Dyson sphere evaporator (DSE-V) [108]	Internal non-evaporative interface heating	4.08	6.00–7.50
8	Fully hydrophilic polymer foam evaporator [222]	Polymer foam, ultralow thermal conductivity	4.32	5.50–7.00
9	Al-based Janus evaporator [223]	Al substrate + laser-engineered microgrooves	2.22	7.50–9.50
10	Spiral-layered hydrogel (SLH) [224]	Graphdiyne (GDY), gradient pore structure	4.23	8.00–11.00
11	Large-scale RO [225]	SWRO membranes + energy recovery	—	0.45–0.80
12	Multi-stage flash (MSF) [226]	Thermal process, cogeneration	—	0.90–2.20

## 6. Performance Evaluation Standards, Practical Application Challenges, and Future Outlook

### 6.1. Standardized Testing Protocols and Reporting Specifications

The remarkable proliferation of novel materials and architectures in SIE research stands in stark contrast to the field’s lack of standardized performance evaluation protocols. This discrepancy creates a significant bottleneck, impeding credible comparison between studies, obscuring genuine technological progress, and ultimately hindering the transition from laboratory discovery to practical implementation. The current landscape is characterized by ad hoc testing conditions and a predominant focus on reporting peak short-term performance under idealized settings, which often yields impressive but misleadingly high efficiency figures and provides little insight into long-term viability. As noted in a recent commentary [208], establishing community-wide “minimum reporting standards” is an urgent priority to ensure scientific rigor, enhance reproducibility, and build a reliable knowledge base for the field’s maturation. Standardization must comprehensively address the entire experimental workflow, encompassing precise control and the reporting of input conditions, adoption of unified calculation methodologies, and the implementation of rigorous, quantitative durability assessments.

The foundation of comparable data lies in the meticulous control and transparent reporting of laboratory testing environments. Critical parameters that are frequently overlooked or underreported—such as the spectral quality and spatial uniformity of the solar simulator, ambient temperature and humidity, airflow, and the precise composition of the feedwater (salinity, pH)—exert a profound influence on evaporation kinetics [227]. For instance, research has demonstrated that ambient humidity alone can alter the measured evaporation rates by more than 50%, rendering comparisons between studies conducted under different conditions largely meaningless. Therefore, conducting tests in environmentally controlled chambers and mandating the full disclosure of these parameters are essential steps. Equally important is standardizing the calculation of key performance metrics, most notably the solar-to-vapor conversion efficiency. The widespread use of the bulk water enthalpy (≈2450 kJ kg^−1^) in efficiency calculations for interfacial systems, where water exists in a highly confined state, has led to the frequent, thermodynamically problematic report of efficiencies exceeding 100% [228].

Beyond initial performance, establishing robust protocols for assessing long-term stability and failure modes is paramount to bridging the lab-to-field gap. Stability claims should be supported by quantitative data from defined durability tests, rather than qualitative descriptions. Proposed standardized assessments include continuous illumination over extended periods (>24 h) to monitor performance decay, cyclic tests simulating day–night operation, and exposure to environmental stressors like high-salinity brine, acidic conditions, or biological contaminants. Crucially, research should diagnose and report the primary failure mechanisms of whether salt crystallization blocking pores, material photo-corrosion, biofilm formation, or mechanical degradation, as this information is invaluable for guiding the design of more resilient systems. In essence, adopting such comprehensive standards is not a constraint on innovation but a necessary foundation for credible advancement. It will enable the community to distinguish incremental improvements under ideal conditions from transformative breakthroughs that genuinely address the intertwined challenges of efficiency, durability, and scalability, which are the critical barriers to real-world application explored in the following discussion [47,229].

To move beyond merely identifying the lack of standardization, we propose a unified testing protocol for SIE systems. The protocol specifies: (i) a solar simulator of Class AAA with AM 1.5G spectrum and spatial uniformity ≥98%, (ii) an irradiance of 1 kW·m^−2^ as the baseline, (iii) ambient temperature of 25 ± 2 °C, (iv) relative humidity of 50 ± 5% for open systems, (v) airflow velocity of 0.5 ± 0.1 m·s^−1^ (or explicitly defined ‘still air’), (vi) feed water salinity of 3.5 wt% NaCl (ASTM D1141-98 for artificial seawater), (vii) a minimum evaporator area of 25 cm^2^, (viii) steady-state defined as <2% variation over 10 min, (ix) evaporation rate based on projected area, and (x) energy efficiency calculated using the temperature-dependent latent heat of vaporization. Adoption of this protocol by the community would enable fair cross-laboratory comparison and accelerate the transition from lab-scale prototypes to practical applications [230].

Beyond these baseline specifications, a critical review must also address common sources of error that can substantially distort reported performance. First, dark evaporation subtraction: the evaporation rate under illumination must be corrected by subtracting the dark evaporation rate measured under identical environmental conditions, yet many studies either omit this step or use inappropriate controls. Second, environmental heat gain: small-scale setups are particularly susceptible to convective and radiative heat from the surroundings, which can artificially inflate the apparent evaporation rate; thermal insulation and careful placement of the setup are essential. Third, evaporator-area normalization: for 3D evaporators, reporting evaporation rate based on the projected area can overestimate performance compared to using the true surface area; the community should adopt a consistent convention (e.g., projected area for 2D and top-surface area for 3D) to enable fair comparisons. Fourth, humidity and airflow effects: even slight variations in ambient humidity or natural convection can change the evaporation rates by >20%; therefore, all reported values must be accompanied by detailed environmental data. Fifth, non-standard solar intensity: while 1 sun (1 kW·m^−2^) is the baseline, real conditions vary widely; studies should also report performance at lower intensities to assess practical viability under cloudy or low-light conditions [1,23].

Most importantly, a fundamental distinction must be made between evaporation flux and actual freshwater yield. The evaporation rate measured by mass loss in an open system reflects the maximum possible vapor generation, but in a real desalination system, a condenser is required to collect liquid water. Condensation efficiency is rarely 100% due to heat transfer limitations, vapor leakage, and droplet retention on condenser surfaces. Consequently, the actual freshwater collection rate can be 20–50% lower than the reported evaporation rate. We therefore strongly recommend that future studies report both the open-system evaporation rate (for material benchmarking) and the closed-system freshwater collection rate (for practical performance evaluation) under identical conditions. Reporting only the evaporation rate without corresponding collection data can be misleading for technology assessment and should be explicitly noted as a limitation [231].

### 6.2. The Gap Between Laboratory Research and Practical Application: A Critical and Multiscale Analysis

The remarkable laboratory achievements in SIE, often characterized by near-perfect efficiencies under one-sun illumination, stand in stark contrast to the sobering realities of field deployment [98]. Bridging this chasm requires a paradigm shift from a singular focus on performance metrics to a holistic embrace of viability metrics. This section conducts a critical, multiscale analysis of the fundamental gaps at the material, system, and techno-economic levels, arguing that overcoming these intertwined challenges is the paramount task for transforming SIE from a compelling scientific phenomenon into a pragmatic technology for global water security [232].

At the material level, durability under continuous outdoor exposure is a primary concern. Most reported photothermal materials—particularly porous hydrogels, biomass-derived carbons, and nanostructured coatings—have been tested only over hours or days in controlled laboratory settings. Under real-world conditions, prolonged UV irradiation can accelerate photo-oxidative degradation of polymers and carbon skeletons, while thermal cycling and repeated hydration–dehydration stress cause mechanical fatigue, cracking, or delamination of the photothermal layer. Moreover, biofouling—the adhesion and growth of microorganisms, algae, or bacteria on the evaporator surface—remains critically understudied. Biofilms not only block water transport channels and reduce light absorption but also introduce biological contaminants into the condensate. The long-term chemical stability of hydrophilic surface modifications (e.g., plasma treatment or polydopamine coatings) in saline or pH-fluctuating water is rarely verified beyond a few weeks. Material leaching, especially of nanoscale fillers or residual catalysts, poses both performance and environmental risks that demand systematic evaluation via accelerated aging tests and leachate analysis. The pursuit of exotic nanomaterials with exquisite optical properties must be tempered by stringent evaluations of cost, scalability, and environmental impact. For instance, while plasmonic gold nanostructures offer unparalleled tunability, their high cost and potential ionic leaching under prolonged operation render them impractical for large-scale use [23]. The field is increasingly turning to carbon-based materials and earth-abundant semiconductors, but these too face the scalability–stability–performance trilemma. Scaling laboratory synthesis to industrial-grade, reproducible manufacturing without compromising the micro/nanostructures critical for performance remains a formidable hurdle. Future material design must prioritize intrinsic stability—through crosslinked networks, protective coatings, or inherently robust architectures—from the outset, rather than as an afterthought.

At the system level, mechanical stability and operational reliability under dynamic outdoor conditions are equally critical. Floating evaporators must withstand wind-induced waves, water-level fluctuations, and potential collisions without structural failure or detachment of the photothermal layer. The adhesive strength between the solar absorber and the underlying substrate or insulation layer has been largely overlooked; delamination after repeated thermal cycles or mechanical disturbance is a common but rarely reported failure mode. In addition, salt management strategies that work well in stationary laboratory tests may fail under real-world conditions where water flow, wind, and temperature are non-uniform. For example, localized salt crystallization at edges or apexes—while effective in lab-scale devices—can become unpredictable in larger modules, leading to uneven salt bridging and the eventual blockage of water pathways. The idealized, static laboratory environment gives way to the dynamic, unforgiving complexity of nature [233]. The performance of a floating evaporator in pure water under constant illumination is a poor predictor of its behavior in real complex water matrices. Oil films can completely deactivate hydrophilic surfaces by blocking water supply, suspended solids can irreversibly clog microchannels, and microbial biofouling can form insulating layers that drastically reduce efficiency. A system’s resilience must be demonstrably tested against this combinatorial assault. Moreover, extreme weather adaptability is non-negotiable [122]. A practical device must withstand diurnal cycles, wind, dust, rain, and seasonal temperature variations without structural degradation or significant performance loss. We therefore call for standardized long-term outdoor testing protocols—spanning at least three months, covering multiple seasons, and including periodic performance monitoring along with post-mortem material characterization—to rigorously assess durability [234].

A practical device must withstand diurnal cycles, wind, dust, rain, and seasonal temperature variations without structural degradation or significant performance loss [235]. Perhaps the most critical yet overlooked metric is the net water yield and energy balance. Laboratory reports glorify evaporation rates, but the freshwater collection efficiency—the fraction of generated vapor that is practically condensed and collected—is often abysmally low due to inefficient condenser design, wind-driven vapor loss, or parasitic heat gains. When auxiliary energy for water pumping, tracking, or forced condensation is accounted for, the net energy gain of some complex systems becomes questionable. Consequently, achieving a low maintenance design that is self-cleaning, salt-rejecting, and durable is paramount for reducing operational costs and enabling deployment in resource-limited settings [236].

### 6.3. Future Frontiers and Breakthrough Directions

The field of SIE now stands at a critical juncture, poised for both theoretical deepening and a paradigm shift. Future breakthroughs will no longer emerge from incremental refinements of existing materials or structures alone, but will fundamentally depend on pioneering advances achieved through deep, interdisciplinary integration spanning next-generation material systems, disruptive physicochemical mechanisms, novel application scenarios, and intelligent methodologies [237]. The core objective of this evolution is to elevate the technology from a highly efficient yet essentially “passive” energy converter into an “active” co-production platform for water, energy, and resources, endowed with autonomous adaptation, multifunctional synergy, and intelligent regulation [238].

The exploration of future materials will extend beyond static photothermal properties toward dynamic intelligence and bio-inspiration. Intelligent responsive materials, capable of reversibly altering surface wettability, pore structure, or optical characteristics in response to environmental cues such as light, heat, salinity, or pH, will enable self-cleaning evaporators, adaptive salt rejection, and on-demand evaporation control [239]. Inspiration will increasingly be drawn from nature: for instance, mimicking the micro/nanostructures of desert beetle shells for efficient water harvesting, or emulating the multi-scale water transport and transpiration mechanisms of plant roots and leaves to optimize thermal and mass transfer pathways [156]. This exploration will be accelerated by high-throughput computation and machine learning, which can screen vast material libraries to predict and design novel composites with tailored photothermal efficiency, specific ion affinity, or stimulus-responsive behavior, dramatically shortening the cycle from molecular concept to experimental validation [27].

A critical future direction is to distinguish between materials that offer realistic scalability and those that remain academic curiosities. While MXenes, covalent organic frameworks, and plasmonic nanoparticles show excellent photothermal performance at the milligram to gram scale, their synthesis for square-meter-scale decentralized desalination is currently economically prohibitive: MXenes require hazardous HF-based etching, COFs demand expensive organic monomers and solvothermal conditions, and noble metals such as gold cost >$60,000 per kilogram. In contrast, biomass-derived carbons (e.g., carbonized wood, coconut char) and conductive polymers (e.g., polypyrrole) can be produced at <$1–10 per square meter using roll-to-roll or pyrolysis methods already established in industry. Therefore, future breakthroughs should prioritize low-cost, scalable materials over exotic nanomaterials unless the latter are accompanied by a clear pathway to large-scale manufacturing and a mandatory cost-per-square-meter estimate [153,240].

At the mechanistic level, breakthroughs in energy efficiency will hinge on more precise control over microscopic interfacial processes and the synergistic use of multiple energy forms. A deeper understanding and engineered creation of the low-evaporation-enthalpy effect in confined aqueous environments such as sub-nanometer channels or hydrated polymer networks could fundamentally reduce the energy required for phase change [130]. Simultaneously, the efficient capture and utilization of non-thermal energy carriers from plasmon decay or semiconductor excited states offer new pathways to circumvent phonon thermalization losses and directly convert light into chemical or electrical energy [241]. The most transformative direction lies in the deliberate coupling of photothermal, electrochemical, and thermoelectric processes. For example, within an integrated device, steam from photothermal evaporation could drive a turbine for electricity generation, with the resulting waste heat and electric power together enabling electrocatalytic pollutant degradation or fuel synthesis, thereby achieving full-spectrum, multi-form value extraction from sunlight [242].

The expansion of application scenarios will liberate the technology from a singular focus on freshwater production toward addressing broader sustainability challenges. This includes developing distributed evaporation systems for soil remediation and precision agriculture, capable of desalinating saline-alkali land, extracting heavy-metal pollutants, and delivering water directly to crop root zones [150]; building robust modular evaporation-crystallization units as the final concentration stage in industrial zero-liquid-discharge wastewater treatment; and designing closed-loop water-recycling devices for life-support systems in space exploration, where their low energy demand and high reliability offer unique advantages in extreme, resource-constrained environments [150]. Permeating all these directions will be artificial intelligence and automation. AI will be deeply integrated across scales: at the material-discovery stage, generative models can propose novel molecular architectures for specific applications; at the system-design stage, optimization algorithms can balance multi-objective trade-offs among evaporation, anti-fouling, and heat transfer to achieve global performance in dynamic environments; and at the operational stage, computer vision and predictive models will enable early fault diagnosis, scaling warnings, and adaptive control, ultimately moving toward unmanned, intelligent, and long-term stable operation [243].

## 7. Conclusions

Over the past decade, solar-driven interfacial evaporation (SIE) has evolved from a foundational demonstration of localized heat conversion into a vibrant, interdisciplinary frontier that bridges materials science, thermal engineering, and environmental technology. This review has traced that evolution, highlighting a decisive paradigm shift underway: the field is moving from a narrow focus on isolated peak performance metrics under idealized lab conditions toward the holistic, application-oriented design of integrated and intelligent systems. Notable progress has been achieved from the advanced development of multifunctional photothermal materials and architecturally refined evaporators for managing water, heat, and salt, to the ambitious integration of co-generation systems that simultaneously harvest water, energy, and resources. Together, these advances reinforce the foundational promise of SIE as a scalable, low-carbon, and decentralized strategy to address pressing global water challenges.

Yet a significant gap remains between compelling laboratory prototypes and reliable, real-world deployment. The primary barrier is no longer evaporative efficiency alone, but a suite of interconnected viability challenges involved in material durability, system resilience under fluctuating environmental conditions, economic feasibility, and full life cycle sustainability. Therefore, the future trajectory and ultimate success of SIE will be determined not by setting new records for evaporation rate under ideal illumination, but by a fundamental reorientation toward solution-driven science. This calls for a deeply integrated co-design philosophy that rigorously accounts for the interdependencies across the material–structure–system–environment continuum from the earliest stages of conception.

To accelerate the transition from laboratory breakthroughs to commercial deployment, the SIE community must confront several critical scientific and engineering challenges. We highlight the most urgent ones below.

1. Standardization of performance metrics. The field lacks a unified testing protocol. We advocate for mandatory reporting under standardized conditions. Evaporation rate, solar-to-vapor efficiency, and freshwater collection efficiency should be reported alongside dark evaporation and environmental parameters.

2. Scalable manufacturing and cost reduction. Most high-performance evaporators rely on lab-scale methods (freeze-drying, electrospinning, hydrothermal synthesis). Developing continuous, roll-to-roll compatible processes and transitioning from noble metals to earth-abundant carbon or polymer-based materials is essential.

3. Durability under real-world conditions. Long-term stability remains understudied. Key issues include salt crystallization under fluctuating sunlight (requiring self-cleaning or directional transport), biofouling on hydrophilic surfaces, delamination or cracking under thermal cycling and mechanical stress, and UV-induced degradation or nanomaterial leaching. Accelerated aging tests and multi-month outdoor validation are urgently needed.

4. System-level integration. High evaporation rates do not guarantee high water yield due to inefficient condensation, vapor leakage, and parasitic energy consumption. Future systems should integrate passive condensation enhancement (radiative cooling, superhydrophobic surfaces), multi-stage latent heat recovery, and for multi-functional systems, net energy balance reporting (e.g., levelized cost of water and energy).

5. Techno-economic and life cycle assessment (LCA). Comparative LCA against reverse osmosis (RO) and membrane distillation (MD) is required to identify realistic deployment niches. Early analysis suggests that SIE is most competitive for small-scale, off-grid, or emergency applications where infrastructure is lacking. Researchers should report cost breakdowns (materials, fabrication, maintenance) and use metrics such as levelized cost of water (LCOW) and energy return on investment (EROI).

This imperative for integrated design unfolds across several critical fronts. At the material level, innovation must extend beyond high optical absorption to equally prioritize long-term chemical stability, scalable and low-impact manufacturing, and inherent resistance to fouling and corrosion. Structurally, designs must evolve from merely enabling evaporation to actively sustaining operation through self-regulating salt rejection, minimized heat loss, and resilience to mechanical stress. At the system scale, integration must be evaluated through the dual lenses of techno-economic analysis (TEA) and life cycle assessment (LCA), ensuring that functional sophistication translates into genuine economic viability and a net-positive environmental footprint. Such systems thinking is essential to bridge the lab-to-field divide.

Looking ahead, the most promising path for SIE lies in evolving from an efficient “evaporator” into an intelligent, distributed water–energy–resource nexus. Convergence with artificial intelligence and machine learning will prove transformative, enabling the predictive design of novel materials, real-time optimization of complex system arrays, and fully autonomous, self-diagnosing operational networks. In this form, SIE can transition from a standalone unit into a versatile platform capable of delivering tailored solutions: supplying independent water security for off-grid communities, enabling precision water management in agriculture and soil remediation, serving as a zero-liquid-discharge module for industrial wastewater, and forming reliable closed-loop cycles for life-support systems in extreme environments.

In summary, solar-driven interfacial evaporation stands at a decisive inflection point. Its potential to contribute meaningfully to water security and the circular economy is clear, yet its real-world impact depends on the global research community’s collective commitment to embedding practicality, durability, and sustainability as core design principles. By shifting focus from a “performance race” in the lab to a “solution race” for the planet, this technology will become mature from a compelling scientific phenomenon into an indispensable pillar of a resilient and sustainable water future.

## Figures and Tables

**Table 1:** Comparison of physical mechanisms, materials, and key features in photothermal conversion.

Mechanism	Primary Materials	Key Process	Spectral Feature	Advantage
Plasmonic Heating	Au, Ag, Al NPs; doped oxides	Non-radiative decay of LSPR	Narrow, tunable peak	High local field enhancement, spectral tunability
Non-Radiative Recombination	Semiconductors (e.g., CuS, Ti_2_O_3_), Carbon materials	Carrier thermalization & non-radiative recombination	Broadband (carbon) or bandgap-dependent	Broadband absorption, high stability, low cost
Molecular Vibrational Heating	Organic Polymers (e.g., PPy, PDA)	Internal conversion & vibrational relaxation	Typically, broad	Good biocompatibility, flexible, facile synthesis

**Table 2:** Comparison of classical and emerging photothermal material families for SIE.

Material Family	Typical Examples	Primary Mechanism	Key Advantages
Plasmonic Metals	Au, Ag, Al nanoparticles	LSPR decay	Spectral tunability, high local field enhancement
Carbon-Based	Graphene, CNTs, biochar	Non-radiative recombination, π-plasmon	Broadband absorption, low cost, chemical stability, scalable
Semiconductors	CuS, Ti_2_O_3_, MXenes, COFs	Non-radiative recombination	Multifunctionality (catalysis, conductivity), tunable bandgap
Polymers/Organic	PPy, PANI, PDA	Molecular vibrational heating	Flexibility, biocompatibility, facile coating/processing
Hybrid Composites	CNT@Metal, polymer/hydrogel-semiconductor	Combined mechanisms	Synergistic properties, multifunctionality, tailored design

**Table 3:** Key material engineering strategies for advanced photothermal evaporators.

Strategy	Core Objective	Representative Methods/Structures	Desired Outcome
Spectral Manipulation	Maximize broadband solar absorption	Plasmonic arrays, photonic crystals, hierarchical porous networks	>95% solar absorptance, minimized reflection & transmission
Surface Engineering	Optimize water supply & vapor escape	Super-hydrophilicity, Janus wettability, gradient porosity	Rapid capillary wicking, low vapor escape enthalpy, anti-salt
Hybridization	Synergize mechanical, optical, thermal properties	Polymer–carbon composites, semiconductor@metal hybrids, hydrogel frameworks	Enhanced strength/flexibility, efficient heat/water transport, stability
Functional Integration	Add non-evaporative value-added functions	Incorporation of catalysts, biocides, thermoelectric/piezoelectric materials	Simultaneous purification, disinfection, or electricity generation

**Table 4:** Comparison of key structural designs for solar-driven interfacial evaporation.

Structure Type	Core Design Principle	Key Advantages	Inherent Limitations	Primary Application Focus
2D Floating	Planar, interfacial heating	Simplicity, low cost, easy fabrication	Limited area, high environmental loss, poor salt resistance	Fundamental material & mechanism study
3D Volumetric	Spatial extension for area & energy gain	High evaporation flux, ambient energy harvesting, reduced optical demand	Increased material use, more complex fabrication, potential structural fragility	High-rate vapor generation, energy-mass coupling studies
Janus Asymmetric	Directional anisotropy in properties	Efficient passive salt rejection, optimized vapor escape, long-term stability	Requires precise control of bilayer interface, can have reduced vapor flux if too hydrophobic	Long-term solar desalination, treatment of hypersaline brine

**Table 5:** Comparison of salt rejection and anti-fouling strategies.

Strategy Category	Core Mechanism	Representative Implementation	Advantages	Challenges/Considerations
Passive Repulsion	Prevents adhesion via inherent properties	Super-hydrophilic/underwater superoleophobic surfaces; ion-exchange hydrogels	Energy-free, simple principle, good for oil repulsion	Can be overwhelmed; does not remove contaminants, only blocks them
Active Expulsion	Controls crystallization location & remove salt	Directed edge/bottom crystallization; gravity-driven brine backflow; microfluidic recirculation	Robust for high salinity, enables long-term operation	Often requires specific structural design; may need minimal flow or geometry management
Dynamic Regulation	In situ property switch in response to stimuli	Light/heat/pH-responsive surfaces (e.g., PNIPAM, spiropyran coatings)	Adaptive, on-demand functionality, potential for self-cleaning	Material complexity, long-term switching stability, integration challenges

## Data Availability

No new data were created or analyzed in this study. Data sharing is not applicable to this article.
